# Chromosome evolution screens recapitulate tissue-specific tumor aneuploidy patterns

**DOI:** 10.1038/s41588-024-01665-2

**Published:** 2024-02-22

**Authors:** Emma V. Watson, Jake June-Koo Lee, Doga C. Gulhan, Giorgio E. M. Melloni, Sergey V. Venev, Rayna Y. Magesh, Abdulrazak Frederick, Kunitoshi Chiba, Eric C. Wooten, Kamila Naxerova, Job Dekker, Peter J. Park, Stephen J. Elledge

**Affiliations:** 1 Department of Genetics, Harvard Medical School and Department of Medicine, Division of Genetics, Brigham and Women’s Hospital, Boston, MA USA; 2 Department of Systems Biology, University of Massachusetts Chan Medical School, Worcester, MA USA; 3 Department of Biomedical Informatics, Harvard Medical School, Boston, MA USA; 4 Massachusetts General Hospital Cancer Center, Harvard Medical School, Boston, MA USA; 5 Center for Systems Biology and Department of Radiology, Massachusetts General Hospital and Harvard Medical School, Boston, MA USA; 6 Howard Hughes Medical Institute, Chevy Chase, MD USA

**Keywords:** Breast cancer, Cancer

## Abstract

Whole chromosome and arm-level copy number alterations occur at high frequencies in tumors, but their selective advantages, if any, are poorly understood. Here, utilizing unbiased whole chromosome genetic screens combined with in vitro evolution to generate arm- and subarm-level events, we iteratively selected the fittest karyotypes from aneuploidized human renal and mammary epithelial cells. Proliferation-based karyotype selection in these epithelial lines modeled tissue-specific tumor aneuploidy patterns in patient cohorts in the absence of driver mutations. Hi-C-based translocation mapping revealed that arm-level events usually emerged in multiples of two via centromeric translocations and occurred more frequently in tetraploids than diploids, contributing to the increased diversity in evolving tetraploid populations. Isogenic clonal lineages enabled elucidation of pro-tumorigenic mechanisms associated with common copy number alterations, revealing Notch signaling potentiation as a driver of 1q gain in breast cancer. We propose that intrinsic, tissue-specific proliferative effects underlie tumor copy number patterns in cancer.

## Abstract (web summary)

This study examines karyotypic selection and evolution in vitro using immortalized mammary and kidney epithelial cell lines, observing aneuploidy patterns specific to each origin tissue that are correlated with frequencies in patient tumors and independent of drivers such as *TP53* mutation.

## Main

Tumors evolve through two primary mechanisms of change: accumulation of nucleotide-level mutations in driver genes and aneuploidy, the gain and loss of large chromosomal regions. Whereas the oncogenic roles of driver mutations have been extensively studied, the functions of chromosomal copy number alterations (CNAs) are poorly understood. Since widespread gene-dosage imbalance and proteotoxic stress are detrimental to cellular function, aneuploidy comes at a cost^1–3^, which seems incompatible with the notion that it is pro-tumorigenic^4–6^. In vitro investigations across species have generally revealed negative effects associated with aneuploidy, with rare exceptions for some specific CNAs that have been shown to provide fitness benefits under stressful conditions^7–9^. Yet, aneuploidy emerges early during tumorigenesis, appearing in pre-cancerous neoplasms^10–12^, increasing in degree as disease stage advances^13–15^. While tumor CNA patterns are tissue-specific^16,17^, common pan-cancer CNAs tend to have skewed distributions of pro-tumorigenic (for example, oncogenes) and anti-tumorigenic (for example, tumor suppressors) genes^18^, suggesting CNAs could promote tumorigenesis through gene dosage of drivers. However, the fitness effects of most cancer-associated CNAs have yet to be examined in experimental models.

Aneuploidy may also promote tumorigenesis via increased genome instability and replication stress, generating more chromosome breaks and structural variation (SV)^19–21^. Whole-genome duplication (WGD) occurs often during tumorigenesis and is associated with intra-tumoral heterogeneity^22–26^, therapeutic resistance and poorer outcomes^27–29^. WGD increases the number of copy number states that chromosomes may adopt and may also buffer against mutation of essential genes^24^. The impact of aneuploidy and polyploidy on cellular fitness and genome evolution in the presence or absence of cancer drivers such as *TP53* mutation is unclear.

In this Article, we utilize unbiased forward genetic screens and in vitro evolution to explore the proliferative effects of chromosomal aneuploidies in human renal and mammary epithelial cells. Cancer-associated CNAs were recurrently selected in culture in a tissue-specific manner, improving growth rates in the absence of classical mutational drivers. Hi-C mapping revealed that centromeric rearrangements facilitated most chromosomal arm-level aneuploidies. Tetraploid cells exhibit increased rates of CNA acquisition, especially centromeric translocation-driven arm-level events, thus supporting a role for WGD in accelerating karyotype evolution during tumorigenesis. Finally, isogenic cell line pairs generated in our screens enabled phenotypic profiling of tumor-associated CNAs, revealing candidate driver genes and pathways. We predict that +1q in breast cancer is driven by Notch signaling through increased expression of 1q-resident γ-secretase genes.

## Results

### Forward genetic whole chromosome copy number screens

**Fig. 1:**
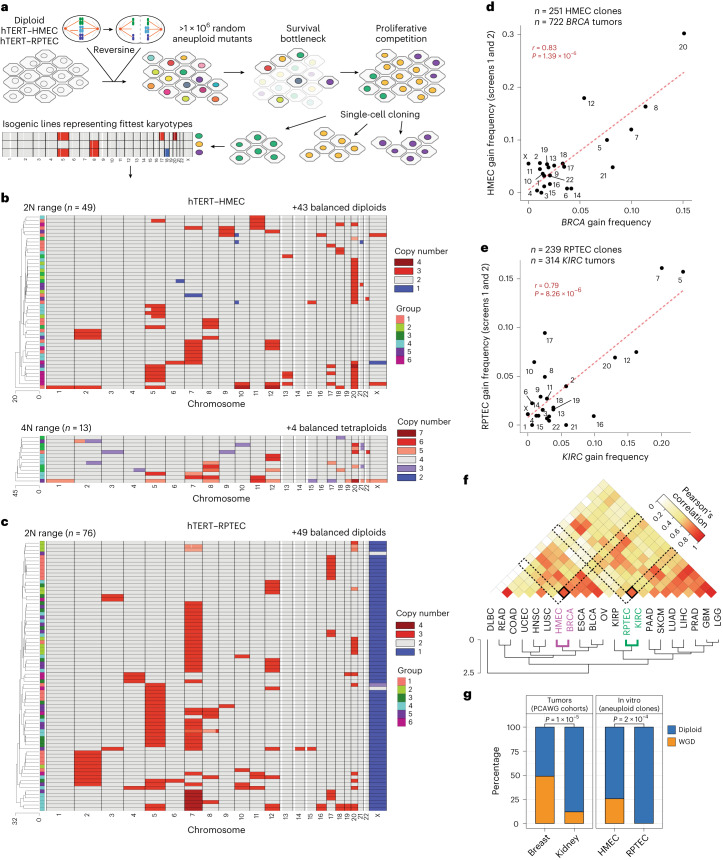
Whole chromosome aneuploidy screens in HMEC and RPTEC cell lines select cognate tumor type whole chromosome CNA patterns. **a**, Whole chromosome aneuploidy screens. Diploid HMECs or RPTECs were treated with reversine (48 h). Cells recovered and proliferated for two PDs (5–7 days), then were cloned, karyotyped by WGS and banked as clonal cell lines. **b**,**c**, Copy number profiles for diploid- (top) and tetraploid-range (bottom) aneuploid clones from the screen for HMECs (**b**) and for diploid-range RPTECs (**c**). Groups indicate independent reversine-treated populations. Red, increased copy. Blue, decreased copy. Gray, neutral copy. **d**,**e**, Frequency correlations of whole chromosome gains in the TCGA breast cancer cohort (all subtypes) and the HMEC screens (**d**), including both diploids and tetraploids (average of two screen replicates), with Pearson’s correlation coefficient (*r* = 0.83) and associated *P* value (*P* = 1.39 × 10^−6^) shown, and RPTEC screens (**e**) with Pearson’s correlation coefficient (*r* = 0.79) and associated *P* value (*P* = 8.26 × 10^−6^) shown in comparison with the TCGA kidney cancer cohort. Dashed red lines indicate linear model fit of the data. **f**, Clustered heatmap of Pearson’s correlation coefficients (*r*) comparing whole chromosome gain frequencies across tumor types and in vitro HMEC and RPTEC screens. DLBC, diffuse large B cell lymphoma; *n* = 47. READ, rectal adenocarcinoma; *n* = 162. COAD, colon adenocarcinoma; *n* = 282. UCEC, uterine corpus endometrial carcinoma; *n* = 425. HNSC, head and neck squamous cell carcinoma; *n* = 522. LUSC, lung squamous cell carcinoma; *n* = 356. BRCA, breast invasive carcinoma; *n* = 722. ESCA, esophageal carcinoma; *n* = 184. BLCA, bladder urothelial carcinoma; *n* = 408. OV, ovarian serous cystadenocarcinoma; *n* = 576. KIRP, kidney renal papillary cell carcinoma; *n* = 272. KIRC, kidney renal clear cell carcinoma; *n* = 314. PAAD, pancreatic adenocarcinoma; *n* = 183. SKCM, skin cutaneous melanoma; *n* = 104. LUAD, lung adenocarcinoma; *n* = 446. LIHC, liver hepatocellular carcinoma; *n* = 370. PRAD, prostate adenocarcinoma; *n* = 420. GBM, glioblastoma; *n* = 521. LGG, low-grade glioma; *n* = 502. **g**, Percentage of the breast and kidney tumors with WGD (PCAWG cohort) (left) and percentage of HMEC or RPTEC clonal cell lines that went through WGD (right). *P* values derived from chi-squared tests. Source data

**Extended Data Fig. 1:**
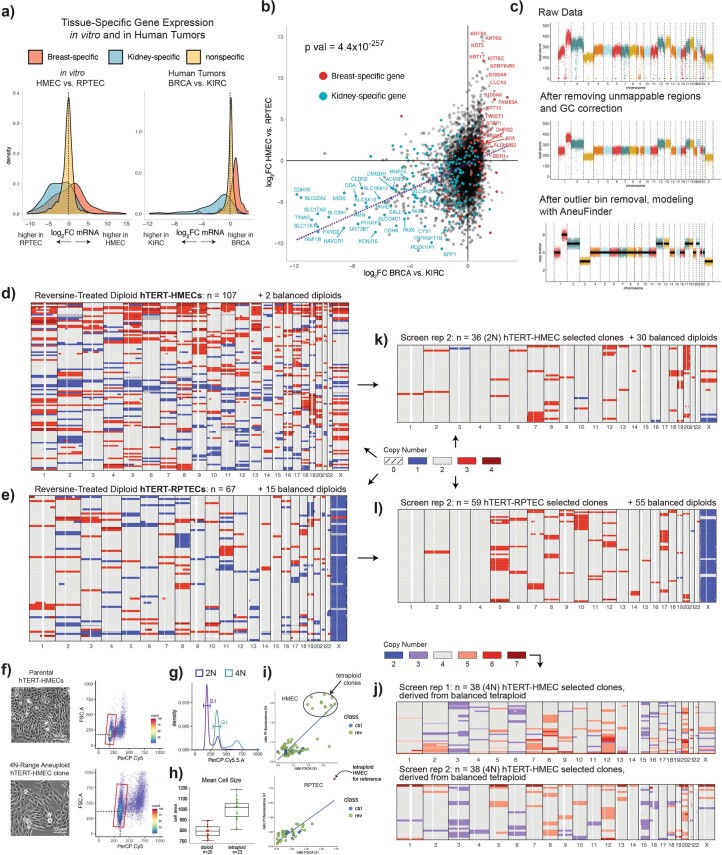
Establishment of aneuploid cell lines from HMECs and RPTECs. (**a**) Distributions of mRNA log_2_FC for Breast-specific (n = 44), Kidney-specific (n = 190), and non-specific (n = 11691) genes in RPTECs vs HMECs (left) and in KIRC vs BRCA tumors (right). RNA-seq data for RPTECs and HMECs generated in this study; RNA-seq data for human tumors is from the TCGA database. Breast- and Kidney-specific genes were annotated by the Human Protein Atlas. **(b**) Scatter plots of mRNA log_2_FC values from the differential expression analysis in (**a**). *P* value (*P* = 4.4 × 10^−257^) calculated from linear regression analysis. **(c**) Low-coverage DNA-seq pipeline for copy number calling. Read counts of raw sequencing data in 100 kb bins is shown after each step of the data analysis pipeline, and final inferred copy number states. **(d**) Single-cell profiles of hTERT-HMECs treated with reversine for 48 hours, clustered by Euclidean distance. **(e)** Single-cell profiles of hTERT-RPTECs treated with reversine for 48 hours, clustered by Euclidean distance. **(f**) Bright field images (left) and propidium iodide staining FACs analysis (right) of the hTERT-HMEC parental population (top) and a tetraploid-range clone (bottom). Gating strategy for G1 population and parameter extraction shown. **(g)** Density plots of PI fluorescence (x-axis) corresponding to scatterplots in (**f**). **(h**) Tetraploid HMEC clones are larger in size than diploid clones based on image analysis from a group of 43 representative clones. **(i)** Mean forward scatter (x-axis) and G1 peak PI fluorescence of HMEC aneuploid clones normalized to parental diploids from both control and reversine-treated populations (top). Tetraploids form a separate cluster. Same is shown for RPTEC clones (bottom; one HMEC tetraploid is included for comparison). **(j**) Copy number profiles of clones selected from HMEC tetraploid screens, replicate #1 (top) and replicate # 2 (bottom), clustered by Euclidean distance. Clone names from this set start with ‘F’ (that is *FA*, *FB*, *FC*, etc.). **(k**) Copy number profiles of diploid HMEC screen replicate #2. **(l**) Copy number profiles of diploid RPTEC screen replicate #2. Source data

To assess selective potentials of various aneuploidies, whole chromosome forward genetic screens were performed in normal diploid human telomerase reverse transcriptase (hTERT)-immortalized human mammary epithelial cells (hTERT–HMECs) and renal proximal tubular epithelial cells (hTERT–RPTECs) (Fig. 1a). These cells recapitulate tissue-specific gene expression patterns (Extended Data Fig. 1a,b) and represent putative cell types of origin for tumor types with distinct patterns of CNAs^16,22,30^. We treated 1.5 × 10^6^ cells in six independent groups with the spindle assembly checkpoint inhibitor reversine^31^ for 48 h to generate pools of aneuploid cells with diverse CNAs (Fig. 1a). The initial aneuploid mutant pool diversity was characterized by single cell DNA sequencing (*n* = 109 reversine-treated HMECs and *n* = 82 reversine-treated RPTECs); all chromosomes were represented in the mutant pool in both gained and lost states with few exceptions, indicating near-saturating aneuploidization (Extended Data Fig. 1c–e). Viable karyotypes competitively proliferated for 6 days (equivalent of two total population doublings of the mutant pool); then single cells were propagated into clonal cell lines.

From these screens, 49 2N-range and 13 4N-range aneuploid HMEC lines, plus four balanced tetraploids (determined via propidium iodide staining), were established (Fig. 1b and Extended Data Fig. 1f–i). The reversine-based screening process was repeated with one balanced tetraploid HMEC clone generating an additional cohort of 38 4N-range aneuploid lines (Extended Data Fig. 1j). In RPTECs, 76 2N-range (and no 4N-range) aneuploid lines were derived (Fig. 1c). Most aneuploidies were whole chromosomal and appeared clonal, indicating karyotypic stability for the ~20 population doublings (PDs) of single cell expansion (Fig. 1b,c). Monosomy was strongly selected against in both screens; 40–50% of events in aneuploid pools were monosomies, whereas monosomies only comprise 1–2% of selected events (Fig. 1b,c and Extended Data Fig. 1d,e), a phenomenon that reflects fitness defects of monosomies in *TP53* wild-type (WT) cell lines^32^. Euploidy is enriched in both cell types (2% euploidy in initial HMECs aneuploid pool enriched to 46% in the selected pool, and 18% euploidy in initial RPTECs aneuploid pool enriched to 42% in the selected pool), consistent with the detrimental effects of most chromosomal aneuploidies.

**Extended Data Fig. 2:**
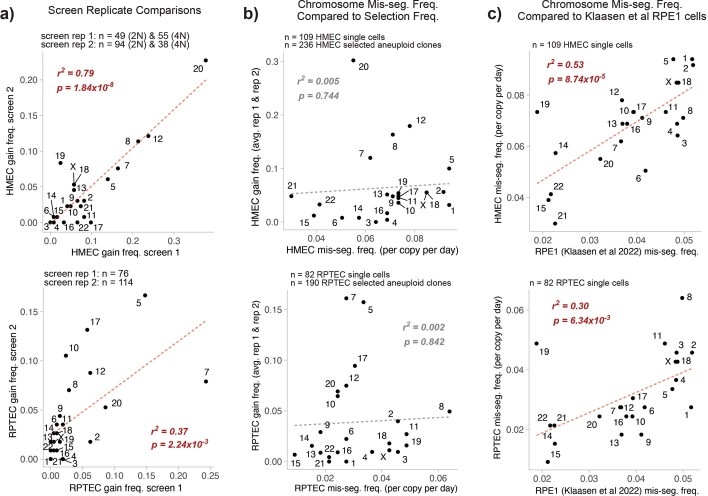
Screen replicate and mis-segregation frequency comparisons. (**a)** Correlation between HMEC screen replicates (top), and between RPTEC screen replicates (bottom) with respect to whole chromosome gain frequency. Pearson’s correlation coefficient squared (top: *r*^*2*^ = 0.79, bottom: *r*^*2*^ = 0.37) and associated *P* value (top: *P* = 1.84 × 10^−8^, bottom: *P* = 2.24 × 10^−3^) are shown. Dashed line indicates linear regression model of the data. **(b)** Top: Correlation between HMEC screen gain selection frequency (average of two screens) and HMEC chromosome mis-segregation frequency with reversine treatment at 48 h. Bottom: Correlation between RPTEC screen gain selection frequency (average of two screens) and RPTEC chromosome mis-segregation frequency with reversine treatment at 48 h. Pearson’s correlation coefficient squared (top: *r*^*2*^ = 0.005, bottom: *r*^*2*^ = 0.002) and associated *P* value (top: *P* = 0.74, bottom: *P* = 0.84) are shown. Dashed line indicates linear regression model of the data. **(c)** Top: Correlation between HMEC chromosome mis-segregation frequency (this study) and RPE1 cell line chromosome mis-segregation frequency (Klaasen et al 2022)^34^. Bottom: Correlation between RPTEC chromosome mis-segregation frequency (this study) and RPE1 cell line chromosome mis-segregation frequency (Klaasen et al 2022)^34^. Pearson’s correlation coefficient squared (top: *r*^*2*^ = 0.53, bottom: *r*^*2*^ = 0.3) and associated *P* value (top: *P* = 8.74 × 10^−5^, bottom: *P* = 6.34 × 10^−3^) are shown. Dashed line indicates linear regression model of the data. Source data

Frequencies of whole chromosome gains were consistent between replicate screens for both lines (Extended Data Fig. 1j–l and Extended Data Fig. 2a), indicating near-saturation of whole chromosome aneuploidization and selection. Selection frequencies are not explained by biases in chromosome missegregation frequencies during initial reversine treatment (Extended Data Fig. 2b), which tend to favor larger chromosomes similar to observations in other cell types^33,34^ (Extended Data Fig. 2c).

**Extended Data Fig. 3:**
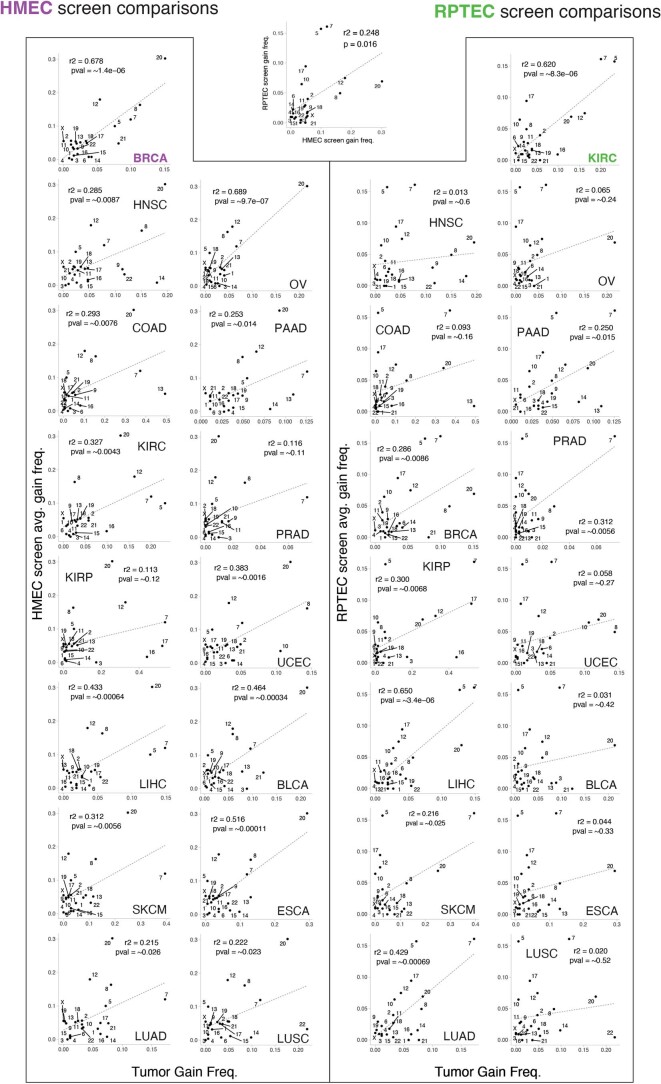
Individual comparisons between in vitro chromosome gain frequencies and human tumor gain frequencies. Corresponding to Fig. 1f. Frequencies of whole chromosome gains in HMEC screens (average of screen 1 and screen 2) compared to various tumor type frequencies (left). The same is plotted for RPTEC screen comparisons on the right. HMEC screen amplification frequencies compared to RPTEC screen amplification frequencies is shown in the top middle panel. Pearson’s correlation coefficient squared (*r*^2^) and associated *P* value are shown. Dashed lines indicate linear regression models of the data. Source data

Selection of whole chromosome gains in the HMEC and RPTEC screens exhibited tissue-type specificity, significantly correlating with incidence rates in their respectively modeled tumor types (breast carcinoma and renal clear cell carcinoma) (Fig. 1d–f and Extended Data Fig. 3). Rates of polyploidy were also significantly different, reflecting the distinct rates of WGD between renal cell and breast carcinomas^35^ (Fig. 1g). These observations suggest that tissue-intrinsic proliferative effects underlie tolerance and/or selection for whole chromosome CNA profiles, as well as WGD.

### In vitro evolution recapitulates arm-level events in tumors

**Fig. 2:**
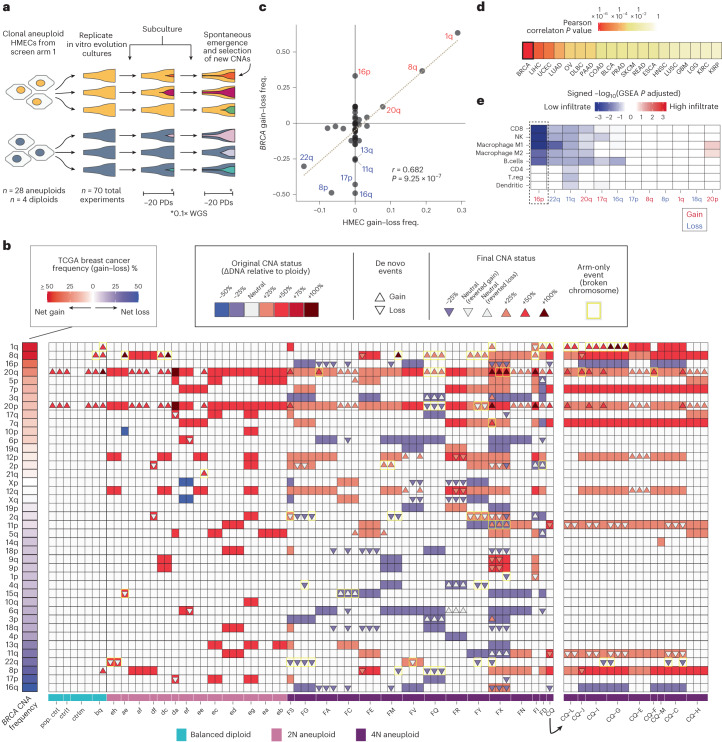
In vitro evolution of aneuploid HMEC lineages leads to convergent selection of breast cancer-associated arm-level CNAs. **a**, Diagram of the in vitro evolution experiments with aneuploid and diploid HMEC clones. **b**, Heatmap summary of all original (first screen, solid squares) and newly selected (triangles) copy number events in long-term evolution experiments in diploid, 2N-range and 4N-range aneuploid HMECs, plotted by arm. The first (wider) column indicates copy number gain or loss frequencies in the breast cancer TCGA cohort. All subsequent columns represent independent in vitro evolution experiments grouped and labeled by clonal lineage and ploidy. Colors inside triangles indicate final copy number state after the newly acquired event. Diploid clone names are indicated by lowercase letters, and tetraploid clones are indicated by uppercase letters. True arm-level events that probably involve broken chromosomes are highlighted in yellow. Right of the heatmap includes a summary of the evolution experiments in daughter subclones of clone *CQ*. **c**, Correlation between true arm-level event frequencies in the HMEC in vitro evolution screen (*n* = 90 in vitro evolution experiments; gain minus loss frequencies) and breast cancer arm-level event frequencies (TCGA cohort, *n* = 722). Pearson’s correlation coefficient (*r* = 0.68) and associated *P* value (*P* = 9.25 × 10^−7^) are shown. Dashed gray line indicates linear regression model of the data. **d**, Heatmap of Pearson’s correlation *P* values from comparisons of chromosome arm-level gain minus loss frequencies in evolved HMECs and various solid tumors (see Fig. 1f legend for tumor type abbreviations and numbers of patient samples). **e**, Transcriptomic GSEA-based immune infiltrate analysis by immune cell type for breast cancers with various CNAs (TCGA database). Gain of 16p (which is not selected in vitro but is frequent in breast cancer) is significantly associated with reduced CD8 T-cell, natural killer (NK) cell and macrophage signatures. *P* values are calculated by assessing the frequency with which the enrichment score of a gene set in a ranking exceeds that of random ranking permutation (10,000 permutations) and is adjusted for multiple gene sets testing. T.reg, regulatory T cells. Source data

**Extended Data Fig. 4:**
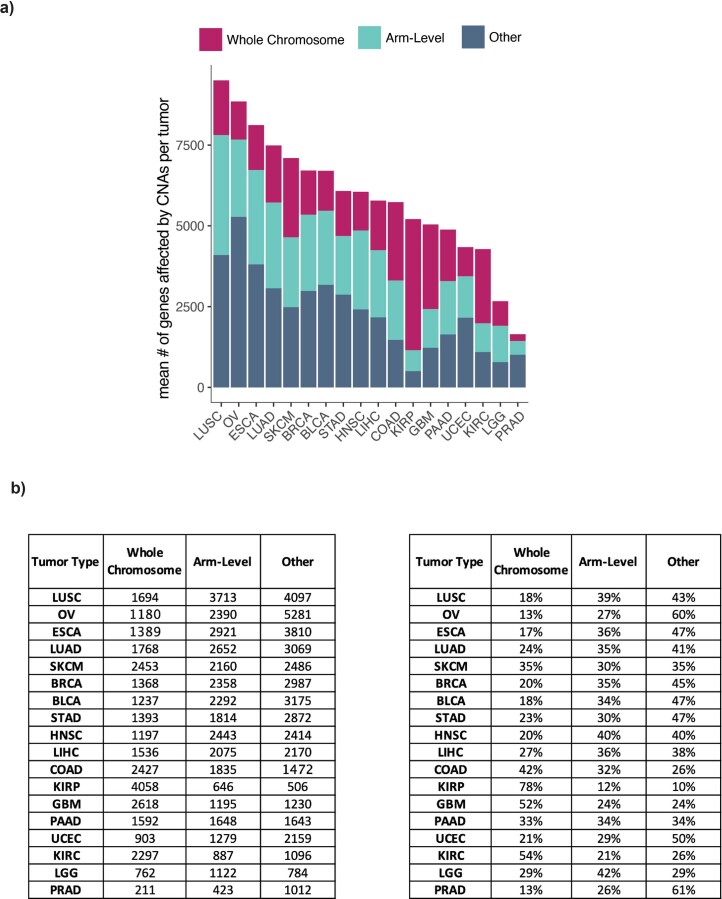
The CNA landscapes of tumors. (**a)** Stacked bar plot showing the average number of genes affected by whole chromosome, arm-level, and all other types of events across various solid tumor types. **(b)** Table showing raw values associated with (**a**), left, and percentages, right, of total number of genes affected by CNAs on average by CNA type. Source data

**Extended Data Fig. 5:**
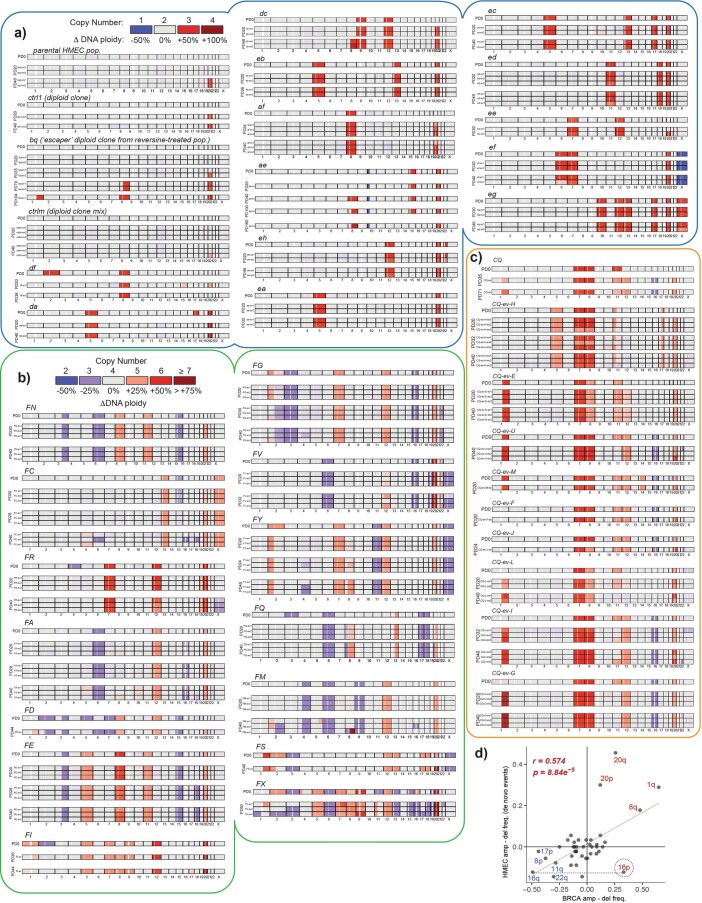
Evolution of clonal HMEC lineages in long-term culture. (**a**) Copy number plots for 2 pure diploid HMEC clones, one diploid clone mix, and 12 2N-range aneuploid HMEC clones grown in culture over time. The top bar of each panel represents the original clonal copy number profile (PD0). Most clones were grown in multiple replicate cultures, for up to 40 population doublings. Several lineages were propagated longer than 40 PDs. **(b**) Copy number plots for 13 4N-range aneuploid HMEC clones grown in culture over time. The top bar of each panel represents the original clonal copy number profile (PD0). Clones were grown in duplicate or triplicate for most lineages, for up to 40 population doublings. **(c**) Copy number plots for *CQ* daughter clone in vitro evolution experiments. Same color bar as for (**b**). **(d**) Net chromosome arm gain/loss frequencies after in vitro evolution experiments (newly selected events only) compared to net gain/loss frequencies in the breast cancer TCGA cohort. Whole chromosome aneuploidies are also counted towards net gain/loss frequencies plotted by arm. For the HMEC frequency calculations, each copy comprising multi-copy events are counted towards the total events, and net event sums are divided by the total number of evolved lineage experiments (n = 90). For breast cancer frequency calculations, at least 50% of the arm must be gained/lost to count as an arm-level event. BRCA; n = 722 samples. Pearson’s correlation coefficient (*r* = 0.574) and associated *P* value (*P* = 8.84 × 10^−5^) for the correlation are shown. Dashed line indicates linear regression model of the data. 16p is highlighted for its opposite behavior in HMECs (deleted as part of whole chromosome 16 loss) and breast cancers (gained), however +16p is associated with immune evasion tumors (see Fig. 2e). Source data

While whole chromosome events contribute appreciably to CNA profiles in tumors (especially in renal cancers), arm-level and subarm-level events are often greater contributors (Extended Data Fig. 4). We therefore executed a second arm of our screen utilizing in vitro evolution to allow aneuploid HMEC clones to spontaneously generate and self-select new CNAs, including arm-level events (Fig. 2a). We performed long-term evolution experiments (35–40 PDs average) with recently expanded HMEC aneuploid clones from the first screen, including 2N- and 4N-range aneuploids, diploid clones and the parental diploid HMEC population, with the majority grown in multiple independent replicate cultures, for a total of 70 experiments (Fig. 2b and Extended Data Fig. 5a,b). A total of 4 of 13 2N-range aneuploid lines and all 15 of the 4N-range lines acquired at least one new CNA in at least one replicate (Fig. 2b and Extended Data Fig. 5a,b). Furthermore, 5 of 13 2N-range aneuploid lines and 9 of 15 4N-range lines reverted one or more CNAs present in their original karyotype back to neutral ploidy (Fig. 2b, white triangles). Most balanced diploid control cultures also gained CNAs over extended time (40–100 PDs), particularly +20, +8q and +1q, which were also frequently selected in aneuploids (Fig. 2b).

Both convergent and divergent karyotypic evolution occurred across replicate cultures of the same clonal lineage (Fig. 2b and Extended Data Fig. 5a,b). To further explore this phenomenon, we derived nine daughter clones from the tetraploid clone *CQ* after it had undergone 35 PDs and further evolved each daughter clone in culture for an additional ~40 PDs. Mother clone *CQ* (++7, ++8 and ++11) evolves +1q, +20, +12 and −16, and reverts +11. True parallel evolution occurred across *CQ* daughter clones, including acquisition of +1q (in three of the six daughter clones that did not already have it), +20 (in the two daughter clones that did not already have it) and reversion of +11 (in four of the eight daughter clones that had not already reverted) (Fig. 2b, right, and Extended Data Fig. 5c).

Of the 127 acquired CNAs across the cohort of evolved HMEC lineages, there were 49 whole chromosome, 74 arm-level and 4 subarm-level events. Arm-level CNA frequencies (affecting one chromosome arm but not the other, indicating a broken chromosome) were significantly correlated with the frequencies of arm-level events in breast cancer (Fig. 2c,d). The most frequent arm-level gains in vitro were 1q and 8q, which are also the most frequent in breast cancer (55% and 50% of cases, respectively). Recurrently lost arms in breast cancer, including chromosomes 8p (51% in patients) and 22q (45%), were also lost frequently during in vitro evolution of HMECs (Fig. 2c). This suggests that selective pressure for acquiring breast cancer-associated CNAs exists inherently in normal mammary epithelia, driven by proliferative effects.

One discrepancy between our in vitro-selected events and the events found in tumors was that HMECs tend to select −16 rather than −16q/+16p (Extended Data Fig. 5d). Interestingly, +16p is associated with reduced immune infiltrate in breast cancer (Fig. 2e). If +16p primarily serves an immune evasion function, its selection may only occur under pressures imposed by the tumor microenvironment^36^, possibly explaining its lack of selection in vitro. Other chromosomes such as −11q may also have immune evasion functions, while −22q may have both pro-proliferative and immune evasion functions.

### Driver gene mutations are not required for CNA selection

**Extended Data Fig. 6:**
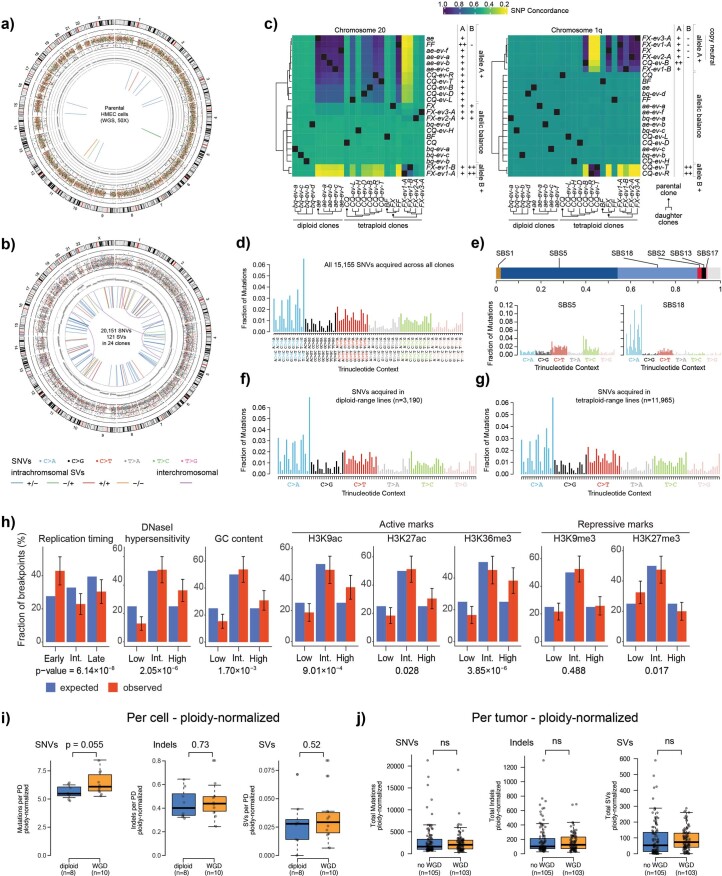
Mutations observed in pre- and post-evolved HMEC lineages. (**a**) A circos plot displaying variants detected in parental HMEC diploid line. Variants were annotated using germline SNP information and those with minor allele frequency greater than 0.001 in human population were filtered out. From outmost to inmost track: chromosomal ideogram, base substitutions with its variant allele frequencies, copy number profile, and structural variations are shown. Detailed mutational information is provided in Supplementary Table 1 − 3. **(b**) A circos plot describing all variants from 24 HMEC clones after in vitro evolution. **(c**) Heatmaps indicating SNP concordance between aneuploid clones analyzed by deep WGS for chromosome 20 (left) and 1q (right). On the x axis, the clones are grouped according to their lineages, which are displayed by dendrograms. Circles on the dendrogram indicates parental clones, and the other branches indicate phylogeny of daughter clones. On y axis, clones were clustered based on concordance of SNP allelic frequencies residing in the chromosomes of interest. Heatmaps were colored using the fraction of shared, amplified SNPs between the clones. Self-comparisons excluded (black squares). **(d**) Spectrum of genome-wide base substitutions in 96 possible trinucleotide contexts across all sequenced HMEC clones. **(e**) Linear decomposition of the observed spectrum using the ICGC/PCAWG-derived mutational signature catalogue. Two mutational signatures related to in vitro culture process explain a large majority of mutations acquired during the evolution. **(f**) Spectrum of genome-wide base substitutions in 96 possible trinucleotide contexts in diploid HMEC clones. **(g**) As in (c) but for tetraploid HMEC clones. Cosine similarity between diploid and tetraploid profiles was 0.986. **(h**) Overlaps of breakpoint positions of acquired SVs with various epigenomic features. We used publicly available epigenomic datasets for the HMEC cell line, except for replication timing dataset which was from the MCF7 breast cancer cell line. To account for the uncertainty of observed values, each error bar is calculated based on a Poisson test. Observed values and their 95% confidence interval are available in Source Data. *P* values derived from goodness of fit test by Chi-square without multiple testing correction. **(i**) Ploidy-adjusted rates of mutations, indels, and non-centromeric SVs in diploid- and tetraploid-range HMECs. *P* values calculated from two-sided Wilcoxon test. **(j**) Ploidy-adjusted counts of mutations, indels, and non-centromeric SVs in breast tumors in the PCAWG dataset. *P* values calculated from two-sided *t*-tests. Source data

During in vitro evolution, acquired non-synonymous single nucleotide variants (SNVs) and structural variants (SVs; insertions, duplications and inversions) affected 193 genes across a subsample of 22 deep-sequenced HMEC clones (Extended Data Fig. 6a,b and Supplementary Tables 1–3). No mutations affected oncogenes (defined by COSMIC^37,38^), and only one potentially damaging mutation affected a tumor suppressor (*AMER1* R358Q; observed in one clone). Two mutations in cancer-related genes were pre-existent in parental HMECs: *NSD1* D588G (unknown significance) and *KMT2D* R5266H (rare germline variant classified as probably benign). None of these genes are considered bona fide drivers in breast cancer^39^. This indicates that mutations in breast cancer-associated tumor suppressors or oncogenes are not required for breast cancer-associated aneuploidies to confer selective advantage in mammary epithelial cells.

### WGD increases karyotypic diversity

**Fig. 3:**
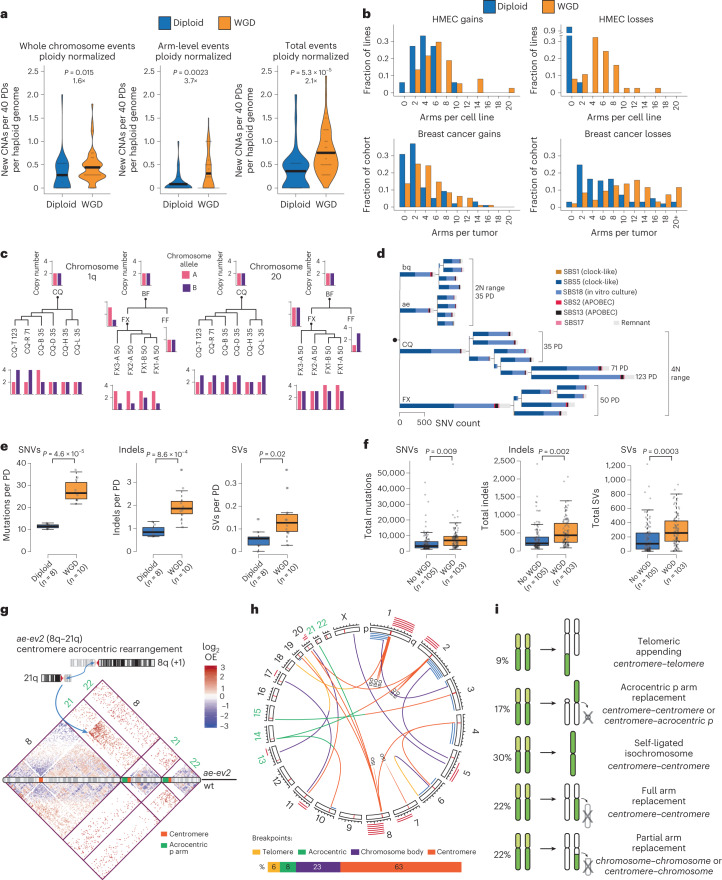
WGD enhances genomic variation. **a**, Ploidy-normalized CNA acquisition rates (per 40 population doublings) in 2N- and 4N-range aneuploids during in vitro evolution. Acquired whole chromosome (left), arm-level (middle) and total (sum of both) (right) events are quantified. *P* values calculated from two-sided Wilcoxon tests. Fold changes of rates between diploid and WGD are also shown. Thick black lines indicate mean rates. **b**, Distributions of arm gains (left) and losses (right) per in vitro-evolved HMEC line (top) and across breast cancers (bottom). Whole chromosome gains/losses are counted as two arms. **c**, Haplotype-resolved CNAs deduced from variant allele frequencies in deep WGS for two evolved tetraploid lineages (*CQ* and *BF*) reveal no absolute allelic preferences for selection of +1q (left) or +20 (right). **d**, Reconstructed phylogenetic tree from shared and private base substitutions in four evolved clonal lineages. Length of branches corresponds to the number of newly acquired base substitutions. Colors indicate mutational signature composition. **e**, Rates of mutation (SNVs), indel and non-centromeric SV acquisition in 2N- and 4N-range HMEC lineages per population doubling (gray dots). *P* values calculated from two-sided Wilcoxon tests. **f**, Total SNVs, indels and non-centromeric SVs in WGD compared to non-WGD breast cancers (gray dots) in the PCAWG dataset. *P* values calculated from two-sided *t*-tests. **g**, Schematic diagram of an acrocentric translocation that mediates gain of 8q through fusion to 21q in clone *ae-ev2*, with 21q remaining neutral (top). Hi-C heatmap of observed/expected values (log_2_ transformed) spanning chromosomes 8, 21 and 22 (upper triangle, *ae-ev2*; bottom triangle, diploid control) (bottom). The blue arrows highlight the fusion event and the corresponding increase in Hi-C contacts. **h**, Circos plot showing all CNA-facilitating translocations detected in this study (top). Individual CNA events are plotted as red (gain) and blue (loss) bars with translocations colored by location (telomere, yellow; acrocentric, green; chromosome body, purple; centromere, orange). Acrocentric chromosomes are labeled with green. Percentages of SV breakpoints involving different chromosomal regions (bottom). ISO, isochromosome. **i**, Schematic diagrams and percentage of occurrences of the five main categories of events observed during in vitro evolution that facilitated arm-level CNA formation. Source data

WGD was associated with significantly more karyotypic events in HMECs, especially arm-level and chromosomal loss events, consistent with observations in human tumors and cell lines^40–42^ (Fig. 3a,b). No allelic preference was observed for selection of CNAs across four evolved lineages (Fig. 3c and Extended Data Fig. 6c). For example, we observe gain of both haplotypes of chromosomes 20 and 1q.

Mutational signatures were similar between diploid and tetraploid lines (cosine similarity of 0.986), dominated by SBS5 (a clock-like signature) and SBS18 (a signature associated with in vitro culture)^43,44^ (Fig. 3d and Extended Data Fig. 6d–g). SVs acquired in vitro were enriched in early replicating regions (Extended Data Fig. 6h), a phenomenon reported in breast cancer^45^. The per cell rates of SNVs, indels and SVs detectable by short-read sequencing in tetraploids were approximately twice that of diploids (Fig. 3e), but largely similar when normalized for total DNA content (Extended Data Fig. 6i). This near-linear scaling of mutational load with DNA content was also observed in human tumors (Fig. 3f and Extended Data Fig. 6j). The doubled per cell SNV, SV and indel rates and the quadrupled CNA acquisition rate all contribute to the increased genetic heterogeneity observed in WGD HMEC lines, and possibly also in WGD tumors^27^.

### Centromeric rearrangements lead to paired CNA events

**Extended Data Fig. 7:**
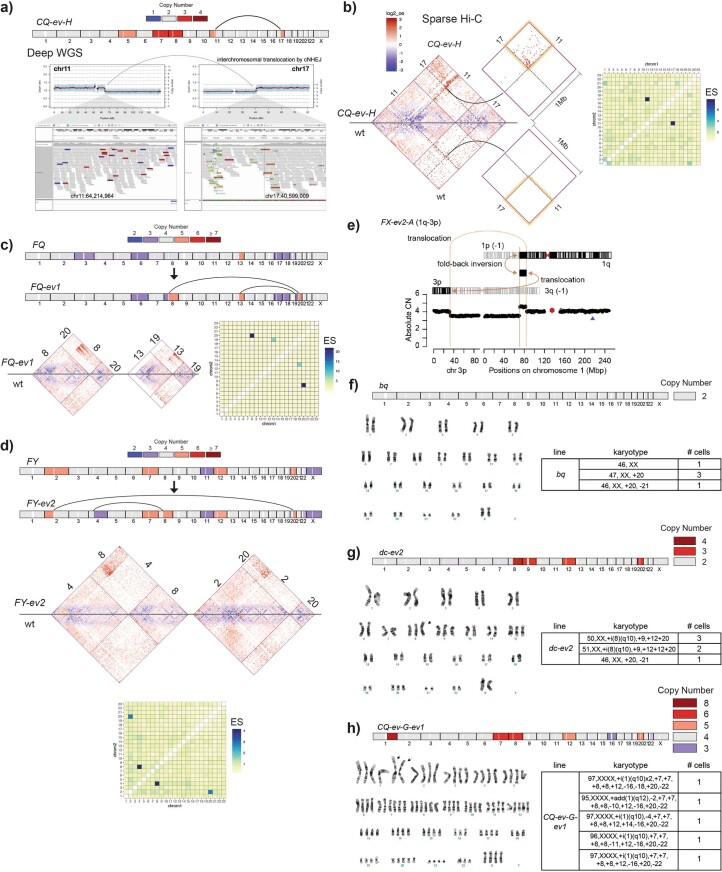
Mapping SVs with low-coverage Hi-C and Giemsa staining. (**a**) Top: Copy number plot for clone *CQ-ev-H*. Bottom: raw read mapping data from deep WGS analysis showing evidence for an 11q-17q translocation breakpoint, which facilitates copy number gains of 11p and 17q. **(b)** Hi-C plots for chromosomes 11 and 17 in the *CQ-ev-H* clone (top triangle of diamond) and diploid control (bottom triangle of diamond). Each pixel represents the log_2_ observed vs expected interaction between a pair of 1 Mb bins (see Methods). Only bins with >1 read are included in the analysis. Since the average number of bin interactions in trans-chromosome interaction space is less than 1, all colored pixels in trans-chromosome interaction space have a positive value. Log_2_ ratios are capped at +3 or −3. The two diamonds to the right are zoom-ins of the 1 Mb region centered on the known translocation, re-binned at 10 kb. The known translocation is indicated by the dotted line. The chromosome 11-17 translocation is automatically detected from Hi-C data by a modified version of the HiNT algorithm (far right panel). ES = enrichment score (HiNT score of mutant/ HiNT score of diploid control). **(c**) Sparse Hi-C mapping of two centromeric translocations in the evolved HMEC *FQ* lineage. **(d**) Sparse Hi-C mapping of a centromeric translocation in the evolved HMEC *FY* lineage. **(e)** Schematic diagram of fold-back inversion identified by deep WGS resulting in an imbalance on chromosomes 1 and 3 in clone *FX-ev2-A*. **(f**) Giemsa staining and karyotyping of the normal diploid HMEC clone *bq*. A karyotype summary of five profiled cells from each is shown on the right. **(g**) Giemsa staining and karyotyping of the evolved 2N-range aneuploid clone *dc-ev2* that gained two copies of 8q. **(h**) Giemsa staining and karyotyping of an evolved 4N-range aneuploid from the *CQ* series that gained 4 copies of 1q. Isochromosomes were suspected based on 1q gain dynamics (occurring in multiples of two) and a lack of evidence of trans-fusions in Hi-C. Copy number plots based on WGS for each line are shown as bars above the G-banding images. Source data

Centromeres and peri-centromeres are known hotspots of CNA boundaries and SVs in tumors^22^, often facilitating recurrent chromosome arm-level aberrations^46^. We generated low-coverage Hi-C maps to efficiently map centromeric translocations in 23 aneuploid clones with arm-level CNAs. As proof of principle, we used this Hi-C pipeline to identify an SV that had been mapped by whole-genome sequencing (WGS) (Extended Data Fig. 7a,b). Although precise centromeric breakpoints could not be mapped with Hi-C, translocations could be detected through increased interaction frequencies between non-neighboring chromosome regions (Fig. 3g, Extended Data Fig. 7b–d and Methods).

Multiple distinct structural mechanisms facilitated arm- or subarm-level CNA formation (Extended Data Fig. 7b–h), 63% of which involve centromeric breakpoints (Fig. 3h,i). These mechanisms include fold-back inversion, fusion to other chromosome arms and isochromosome formation. Most arm-level CNAs occur via paired events, either in *cis* though isochromosome formation (two CNAs affecting the same chromosome arm, fused to itself) or in *trans* though hybrid chromosome formation consisting of two arms from different chromosomes (Fig. 3h,i). Occasionally CNAs appeared as ‘solitary’ events, and we found that these involved either appendage of the gained chromosomal regions to telomeres or, more commonly, fusion to an acrocentric chromosome, possible by replacing acrocentric p arms (Fig. 3h,i). Whether repetitive non-coding regions may be lost such as telomeric regions or acrocentric p arms could not be determined with our methods. In conclusion, most arm-level CNAs emerge as paired events through centromeric translocations.

### Karyotypic evolution mitigates general aneuploidy stress

**Fig. 4:**
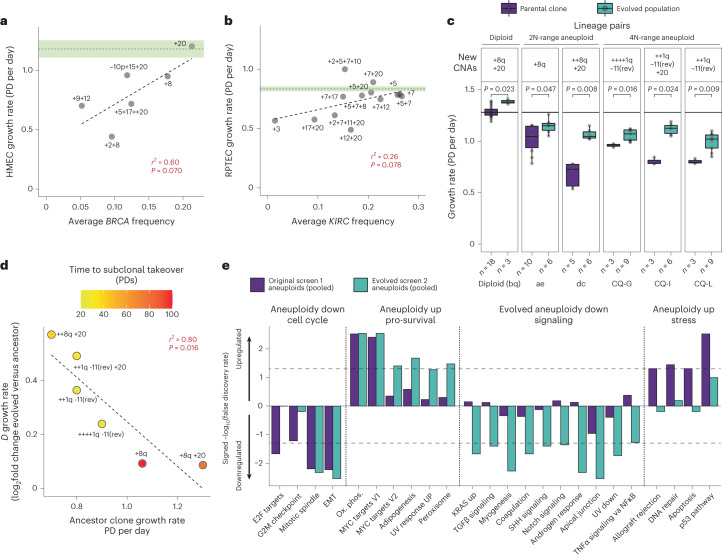
Multiple cancer-associated aneuploidy events can significantly improve growth rate. **a**,**b**, Correlation of growth rates (PDs per day) for HMEC (**a**) and RPTEC (**b**) aneuploid clones compared to average CNA frequencies in cognate tumor types, breast cancer (**a**) and renal cancer (**b**). Parental diploid population growth rates are indicated by horizontal dotted black lines, ± standard error of the mean (green shading). **a**, Pearson’s correlation coefficient squared (*r*^2^ = 0.60) and associated *P* value (*P* = 0.070) are shown. Dashed line indicates linear regression model of the data. **b**, Pearson’s correlation coefficient squared (*r*^2^ = 0.26) and *P* = 0.078. **c**, Growth rates of evolved lineages that gained combinations of +20, +8q and/or +1q compared to pre-evolved isogenic ancestors. *P* values calculated from two-sided Wilcoxon tests. Solid black line indicates median diploid control growth rate. rev, reversion. **d**, Correlation of the growth rate differences (*D*, delta) between evolved and ancestor clones, relative to ancestor clone growth rate. Colors indicate time to clonal sweep of CNAs (in PDs). Plus signs indicate copies gained (one plus sign, one copy). Pearson’s correlation coefficient squared (*r*^2^ = 0.80) and associated *P* value (*P* = 0.016) are shown. Dashed gray line indicates linear regression model of the data. **e**, Bar graphs showing Hallmark GSEA scores (signed −log_10_(false discovery rate)) of differentially expressed gene sets in newly aneuploidized (pre-evolved) clones (dark blue) and in post-evolved aneuploids (light blue), each compared to diploid controls. All copy number-specific effects on gene expression were normalized before analysis. Gene sets with differential expression are grouped on the basis of their relative behavior in pre- and post-evolved aneuploid cells. EMT, epithelial-to-mesenchymal transition; Ox. phos., oxidative phosphorylation; UV, ultraviolet. Source data

Whole chromosome HMEC and RPTEC aneuploid cell lines displayed a range of growth rates, which were often reduced compared to diploids (Fig. 4a,b), consistent with previous findings that aneuploidy reduces fitness^1,3,47^. However, clonal growth rates correlated with the average frequency of their whole chromosome CNAs in cognate tumor type cohorts (Fig. 4a,b). Within evolved lineages, HMECs that gained +8q, +20 and/or +1q had significantly improved growth rates compared to parental ancestor clones (Fig. 4c). The magnitude of growth rate improvements correlated with ancestor clone fitness; acquired CNAs provided more benefit to less fit ancestors and less benefit to more fit ancestors (Fig. 4d). This may explain differences in time to clonal sweep of CNAs in various lineages (Fig. 4d).

**Extended Data Fig. 8:**
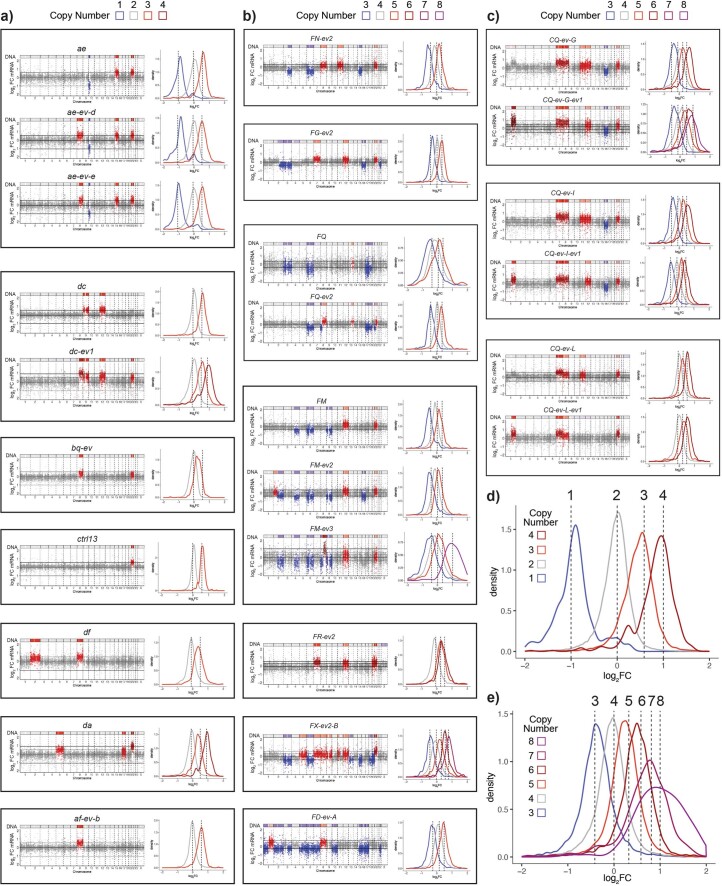
RNA-seq analysis of HMEC diploid- and tetraploid-range aneuploid cell lines. (**a**) Gene expression is directly related to copy number, as shown by mRNA log2 fold changes (log_2_FC) of ten 2N-range aneuploid cell lines compared to control diploids (three replicates per line). Each dot is a gene ordered by genomic position and colored according to the known DNA copy number, with DNA copy number profiles above each plot for reference. The distribution plots to the right of each panel indicate the log_2_FC in mRNA levels for all genes representing each ploidy state in the aneuploid cell line. Lines indicate where mRNA expression would be expected if totally concordant with DNA log_2_FC from baseline ploidy. Clones from the same aneuploid lineage are boxed together (that is ancestor clone and evolved population). **(b**) Gene expression plots as in a), but for 4N-range aneuploid clones. **(c**) Gene expression plots for several *CQ* lineage daughter clones, pre- and post-evolved (top and bottom plots in each box). **(d**) Summary of all RNA-seq data for 2N-range aneuploid HMEC clones in (**a**) normalized to diploid controls. Log_2_FC distributions of genes on chromosomes with copy number 1, 2, 3, or 4. **(e**) Summary of all RNA-seq data for 4N-range aneuploid HMEC clones in (**b-c**) normalized to diploid controls. Log_2_FC distributions of genes on chromosomes with ploidies 3, 4, 5, 6, 7, or 8. Source data

We profiled the transcriptomes of 26 aneuploid HMEC lines, including pre- and post-evolved cultures from seven clonal aneuploid lineages (two 2N-range and five 4N-range pre- and post-evolved pairs) as well as four diploid control clones. Expected CNA-dependent gene expression changes were observed for each clone (Extended Data Fig. 8a–c), and aggregate data indicated little-to-no dosage compensation of CNA-driven transcriptomic effects (Extended Data Fig. 8d,e). Gene set enrichment analysis (GSEA)^48^ revealed a stress signature in pre-evolved, mostly whole chromosome aneuploid HMECs compared to diploids, including increased TNFα/NFκB, inflammation, ROS, p53 and apoptosis pathways (Fig. 4e). These stress signatures were reduced after karyotypic evolution and acquisition of breast cancer-associated CNAs (Fig. 4e). Thus, karyotypic refinement via acquisition of breast cancer-associated CNAs mitigated aneuploidy-associated stress and conferred proliferative advantage.

### Top cancer-associated CNAs increase diploid growth rate

Aneuploidy stress mitigation alone cannot completely explain all effects of breast cancer-associated CNAs on growth rate, since +20 and +8q also conferred a small (5%) but significant growth rate advantage in diploid cells (Fig. 4c). Selection of +20 occurred in multiple independent diploid clones (Fig. 2b). Likewise, RPTEC + 5 and +5 + 20 cells exhibited near-diploid growth rates and some aneuploid clones proliferated faster than diploids (Fig. 4b). Thus, while stress mitigation plays a role in karyotypic refinement in cells that are already aneuploid, general pro-proliferative effects can drive selection of cancer-associated CNAs in diploids, even in *TP53*–WT backgrounds.

### Gain of 8q is associated with a MYC activation signature

**Fig. 5:**
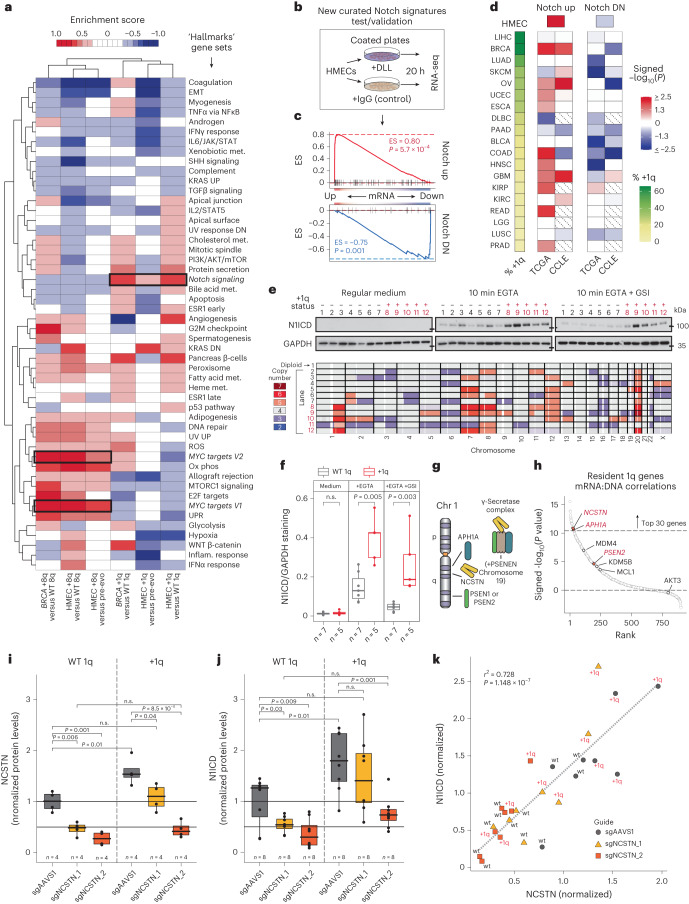
The +1q is associated with increased Notch activation in vitro and in human tumors due to increased γ-secretase gene dosage on 1q. **a**, Hallmark GSEA profile clustering of +1q or +8q HMECs and breast cancers relative to WT counterparts. MYC and Notch gene sets outlined in black. ROS, reactive oxygen species; met., metabolism; DN, down; UPR, unfolded protein response; Inflam., inflammatory; EMT, epithelial-to-mesenchymal transition; Ox phos, oxidative phosphorylation; UV, ultraviolet. **b**, Diagram of ligand-based Notch activation assay. DLL, DLL1 + DLL4 recombinant protein. **c**, Curated Notch activation and repression gene set enrichment in HMECs after 20 h ligand exposure. GSEA scores (0.80 and −0.75) and associated *P* values (*P* = 5.7 × 10^−4^ and *P* = 0.001) are shown, respectively, for Notch activation (up) and repression (DN) sets. **d**, Signed −log_10_ *P* values from GSEA with curated Notch gene sets for +1q versus WT 1q differential expression rankings across tumor types, sorted by prevalence of +1q. Cancer types with <10 samples in the CCLE^77^ not included (hatched squares). DN, down. **e**, Western blots (top) showing cleaved NOTCH1 (N1ICD) in +1q and WT 1q HMEC lines (bottom) in response to calcium depletion (4 mM EGTA, 10 min), ±GSI (5 μM L-685,458, pre-incubated for 30 min). GAPDH shown as loading control. **f**, Quantification of N1ICD (**e**), normalized to GAPDH. *P* values calculated from two-sided Wilcoxon test. **g**, Diagram of the γ-secretase complex and gene locations on 1q. **h**, Ranked 1q-resident gene mRNA/DNA correlations (signed −log_10_ *P* value from Pearson’s correlations) in matched tumor/normal BRCA samples. γ-secretase genes labeled red. Other proposed drivers of 1q are also shown. **i**,**j**, Western blot quantification of NCSTN protein (**i**) and N1ICD (**j**) in diploid (WT 1q) and +1q HMECs infected with lentivirus containing either control (sgAAVS1) or *NCSTN*-targeting CRISPR guides and treated with EGTA for 10 min. NCSTN and N1ICD levels were normalized to GAPDH, and NCSTN/GAPDH and N1ICD/GAPDH ratios were normalized to the average ratio of sgAAVS1 WT 1q cells. *P* values were calculated from two-sided *t*-test, not corrected for multiple testing. n.s., not significant. **k**, Correlation between NCSTN and N1ICD protein levels in each sample quantified in **i** and **j**. Pearson’s correlation coefficient squared (*r*^*2*^ = 0.73) and associated *P* value (*P* = 1.15 × 10^−7^) are shown. Dashed line indicates linear regression model of the data. Source data

**Extended Data Fig. 9:**
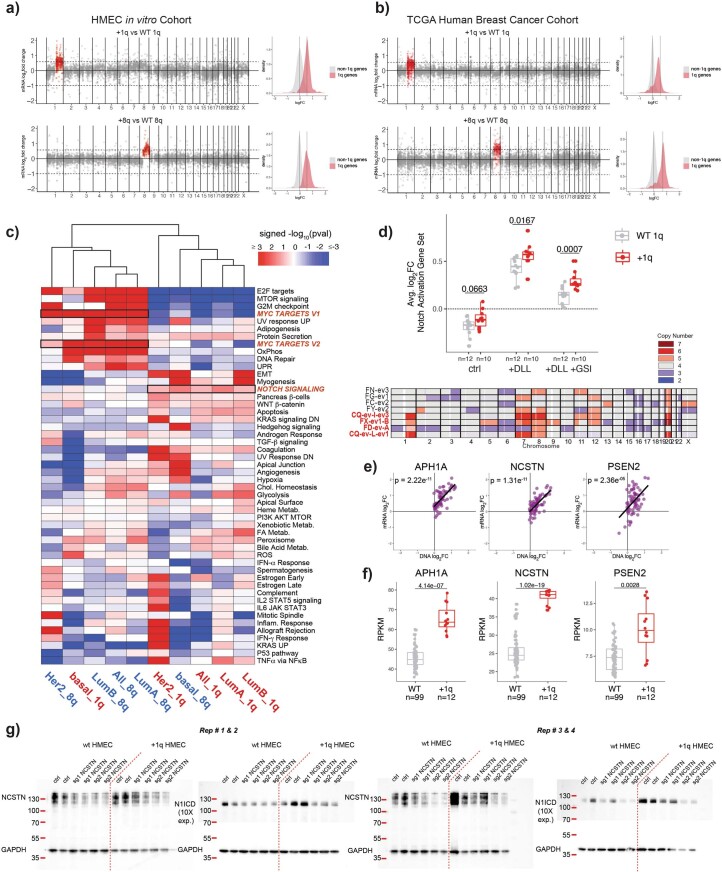
+1q and +8q associated gene expression changes in HMECs and breast tumors. (**a**) HMEC lines were grouped according to +1q (top) or +8q (bottom) status and differential gene expression analysis was performed. mRNA log_2_ fold changes are plotted for all expressed genes across the genome. Panels on the right show the distributions of log_2_FCs for resident genes on 1q (top) or 8q (bottom) compared to all other genes. **(b**) Same analysis as in **(a)** but for TCGA breast cancer samples. **(c**) Gene set enrichment analysis of +1q and +8q tumors in each major breast cancer subtype, and across the entire cohort (‘All’). Genes were ranked based on their differential expression in +1q or +8q tumors within each subtype. The Hallmarks gene sets were used. Colors indicated signed negative log_10_ *P* values from GSEA. **(d**) Top: +1q or WT 1q HMECs were exposed to ligand (DLL1 + DLL4 combined 2.5 µg/ml + fibronectin, coated plates), or ligand + GSI (2 μM L-685,458) for 20 h. Control plates (no ligand) were coated with 2.5 µg/ml human IgG + fibronectin. RNA-seq analysis was performed and average log_2_FC of Notch Activation gene set is plotted for each condition relative to diploid control conditions. +1q HMECs display increased Notch activation capacity when incubated for 20 h on ligand-coated plates, and increased residual Notch activation when GSIs are added. *P* values calculated from two-sided *t*-test. Bottom: WT 1q and +1q cell lines used in this experiment. **(e**) Correlation between mRNA log_2_FC and DNA log_2_FC in matched tumor-normal breast cancer TCGA data for the three γ-secretase genes on 1q. *P* values calculated from linear regression analysis. Dashed lines indicate linear regression models of the data. **(f**) Expression levels for resident 1q γ-secretase genes *APH1A*, *PSEN2*, and *NCSTN* in +1q and WT 1q HMECs. *P* values calculated from two-sided *t*-test. **(g**) A total of four replicate experiments were performed comparing NCSTN knockdown in WT 1q and +1q HMEC lines. NCSTN (first and third panels) and N1ICD (second and fourth panels) were blotted from lysates of cells treated with EGTA for 10 min. N1ICD imaging required 10x longer exposure. Source data

We analyzed gene expression with respect to +8q in aneuploid HMECs and human breast cancer samples. In addition to strong positional enrichment of differentially expressed genes along 8q (Extended Data Fig. 9a,b), a similar Hallmark gene set enrichment profile characterized by increased MYC signaling was observed in vivo and in vitro (Fig. 5a and Extended Data Fig. 9c). *MYC* is a resident gene on 8q and is known to be one of the most potent drivers of HMEC proliferation^17^. Our data indicate that shallow gain of the entire 8q arm is sufficient to upregulate MYC signaling in mammary epithelial cells. In breast cancer, focal *MYC* amplification is relatively rare (~6% in The Cancer Genome Atlas (TCGA) and the International Cancer Genome Consortium cohorts), whereas arm-level amplification of 8q is common (~50%). Since gain of only one or two copies of 8q results in strong MYC signature activation, *MYC* probably contributes to the selective advantage of +8q.

### Gain of 1q is associated with increased Notch signaling

The functional impact of +1q, the most frequent genomic alteration in breast cancer (55–60% of patients), is more enigmatic, although some candidate drivers such as *MDM4* (ref. ^49^), *MCL1* (ref. ^50^), *AKT3* (ref. ^51^) and *KDM5B*^52^ have been proposed. Breast cancers usually amplify the entire arm without minimal consensus segments. Competing +1q subclones were observed during HMEC evolution (Fig. 3c), a phenomenon also observed in single-cell and multi-region tumor sequencing^53,54^, and even in adjacent normal tissues^55^. We analyzed the transcriptomes of +1q HMECs and +1q breast tumors (Extended Data Fig. 9a,b) and found that the most consistently upregulated pathway is the Notch juxtracrine cell-patterning system (Fig. 5a and Extended Data Fig. 9c).

Notch controls ductal branching during mammary development; loss-of-function mutations lead to branching failure and mammary gland defects^56,57^, whereas Notch gain-of-function mutations lead to hyper-branching, hyperplasia and eventually tumor formation^58–62^. Given that activating Notch mutations occur in ~5% of breast cancers^63,64^, Notch is considered an oncogene in mammary epithelia.

We curated high-quality Notch activation and Notch repression gene signatures from previously published Notch overexpression, knockdown and inhibitor RNA sequencing (RNA-seq) experiments^65,66^, as well as Notch intracellular domain (NICD) chromation immunoprecipitation^67^ and pulldown mass spectrometry^68^ datasets (Supplementary Table 4). Notch signatures were validated by incubating HMECs with ligand-coated plates (recombinant DLL1 + DLL4) for 20 h, which strongly activated the Notch activation signature (117 genes) and repressed the Notch repression signature (34 genes) (Fig. 5b,c). Across various tissue types, +1q tumors (TCGA) and +1q cancer cell lines (Cancer Cell Line Encyclopedia (CCLE)) exhibit significantly increased Notch activation signatures and decreased Notch repression signatures (Fig. 5d).

To directly measure Notch activation capacity in response to transient activation signal (10 min Ca^2+^ depletion, which dissociates the Notch extracellular domain^69,70^), we utilized a cleaved NOTCH1-specific antibody. The +1q HMECs activated approximately 2.2-fold more Notch than WT 1q HMECs (Fig. 5e,f). γ-secretase inhibitor (GSI) pre-incubation was sufficient to prevent EGTA-induced Notch cleavage in WT 1q HMECs, and partially in +1q HMECs. This +1q phenotype was also observed when cells were incubated with activating DLL ligand (Extended Data Fig. 9d).

Notch signatures are not significantly enriched for 1q-resident genes (*P* = 0.112, two-tailed chi-squared test); however, three γ-secretase components reside on 1q: *APH1A*, *NCSTN* and *PSEN2* (ref. ^71^) (Fig. 5g). All three genes were significantly upregulated in +1q HMEC cell lines and +1q breast tumors (Fig. 5h and Extended Data Fig. 9e,f), particularly APH1A and NCSTN.

We used clustered regularly interspaced short palindromic repeats (CRISPR)-mediated gene editing with two different single guide RNAs to partially knock out *NCSTN* in +1q cell populations to baseline WT or below baseline levels. We generated a spectrum of NCSTN expression levels in a range relevant to the differential expression between WT and +1q levels (Fig. 5i and Extended Data Fig. 9g). The increased Notch activation capacity observed in +1q HMECs directly depends on the increased gene dosage of *NCSTN* (Fig. 5j and Extended Data Fig. 9g). Across the spectrum of editing efficiencies in +1q and WT cells, NCSTN was highly correlated with cleaved Notch abundance (Fig. 5k), indicating that NCSTN/γ-secretase levels largely dictate Notch activation capacity and are responsible for increased Notch signatures in +1q HMECs.

### A Notch-poising mechanism may drive +1q selective advantage

**Fig. 6:**
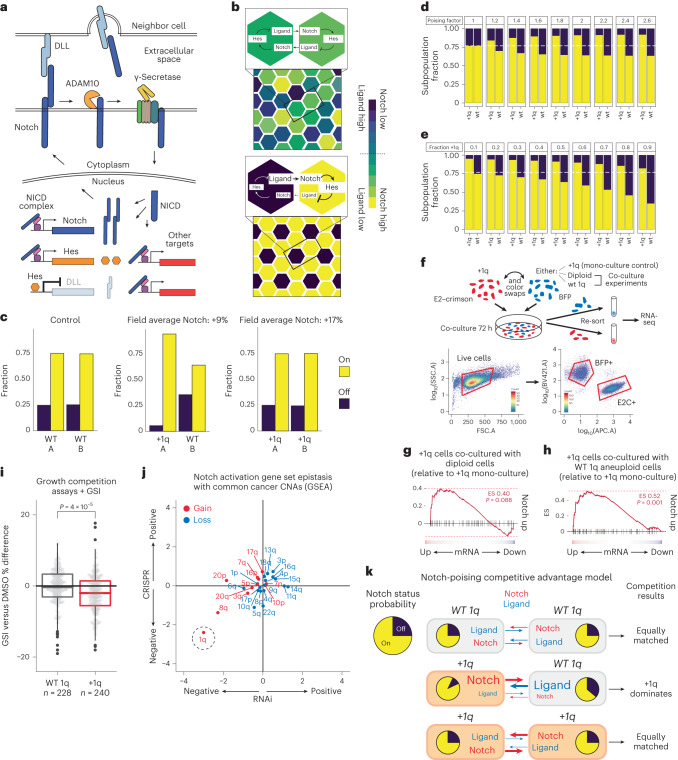
A model for +1q-driven Notch poising. **a**, Diagram of the Notch signaling pathway. **b**, In silico simulations of Notch lateral inhibition in a 40 × 40 field of cells (see Supplementary Video 1). The simulation starts with randomly assigned Notch status (top) and is run over 1,000 min, which generates a Notch-on/Notch-off pattern across the field of cells (bottom). **c**, Results of simulations of mono-cultured WT (left), +1q (right) and co-cultured (middle) populations with respect to Notch activation status. Cells are randomly assigned to group A or B. The fraction of cells after simulation with N1ICD >0.5 (on) and N1ICD <0.5 (off) for groups A and B is shown. WT 1q poising factor = 1. +1q poising factor = 2.2 (based on experiments in Fig. 5e). **d**, Simulations varying the Notch-poising factor. **e**, Simulations varying the proportion of +1q and WT 1q co-cultured subpopulations. **f**, Co-culture experimental design to test dominant lateral inhibition as predicted by modeling (top). Gating strategy for sorting BFP- and E2–crimson-tagged populations after co-culture (bottom). **g**,**h**, Expression of the Notch activation signature in +1q HMECs co-cultured with diploid HMECs compared to mono-cultured +1q HMECs, with GSEA score (0.40) and associated *P* value (*P* = 0.088) (**g**), and co-cultured with WT 1q aneuploid HMECs with GSEA score (0.52) and *P* value (*P* = 0.001) (**h**). **i**, The fraction of viable WT 1q (blue) or +1q (red) cells in co-culture with WT 1q cells ± GSI (2 μM L-685,458, 72 h). DMSO, dimethyl sulfoxide (control). **j**, DepMap analysis of epistasis between common arm-level CNAs and the Notch activation gene set, in RNAi (*x* axis) and CRISPR (*y* axis) datasets. Genes were ranked based on their effect score correlation to CNA status. GSEA was then performed for each CNA-based epistasis ranking using the Notch activation signature. Cancer cell lines derived from tumor types with high frequencies of +1q were used for this analysis (breast carcinoma, lung adenocarcinoma and liver hepatocellular carcinoma). **k**, Model for +1q-driven Notch poising and increased juxtracrine competition. As +1q subclones encounter WT 1q cells, they occupy mostly Notch-on states, providing growth advantage. Source data

Notch signaling initiates through binding to ligand (DLL or JAG) expressed on the surface of neighboring cells; then γ-secretase-cleaved Notch translocates to the nucleus and activates both itself and repressors (HES or HEY) of its own ligands (Fig. 6a). By repressing its own ligands, Notch-activated cells starve their neighbors of ligand, thus preventing neighbors from activating their own Notch and therefore coaxing them to produce more ligand. This feed-forward ‘lateral inhibition’ leads to a stable bifurcation of Notch-on/off states in a spatially alternating pattern (Fig. 6b). Since our experiments revealed that +1q HMECs have the capacity to activate approximately twofold more Notch than WT 1q cells in response to transient signal, but only display a modest increase in activated Notch at steady state or under ligand-saturating conditions (Extended Data Fig. 9d), we hypothesized that +1q poises Notch for activation rather than constitutively activates it—potentially providing a competitive advantage under ligand-limiting or competitive juxtracrine situations.

To explore this hypothesis we utilized an in silico model of Notch lateral inhibition^72^, in which ‘Notch-poised’ +1q cells can activate twofold more Notch in response to neighbor-provided ligand (Fig. 6b and Supplementary Video 1). Simulations revealed that pure +1q or pure WT 1q cell populations achieve the same ratios of Notch-on:Notch-off cells (3:1) once at steady state (although pure +1q populations displayed marginally higher field-average levels of activated Notch since Notch-on cells were likely to be maximally activated) (Fig. 6c). Simulation of a well-mixed co-culture of +1q (poised) and WT 1q (non-poised) cells resulted in a skewed population: +1q cells were enriched for Notch-on status, while WT 1q cells were enriched for Notch-off status (Fig. 6c and Supplementary Video 1). Therefore, +1q-driven Notch poising may be most beneficial when cells are in contact with WT 1q neighbor cells in mixed populations by tipping the balance of lateral inhibition.

The predicted benefit of Notch poising in mixed culture peaks at a poising factor of ~2 (Fig. 6d), which is approximately what is observed for +1q in vitro. Another implication of this model is that the benefits of poising depend on the number of contacts between non-poised and poised cells, such that poised cells at low concentrations are constitutively Notch-activated because they physically contact mostly non-poised cells (Fig. 6e). Therefore, +1q subclones in physical contact with majority WT 1q tumor cells may experience the strongest competitive advantage.

**Extended Data Fig. 10:**
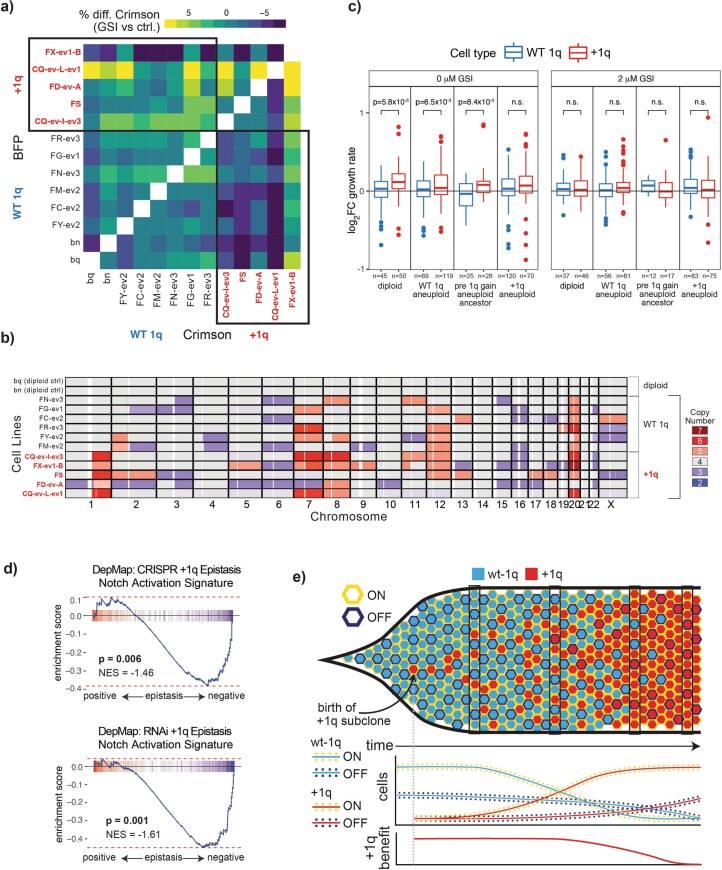
+1q mediated Notch poising. (**a**) Matrix of clone vs clone co-culture +/− GSI experiments summarized in Fig. 6i. Average of three biological replicate experiments is shown for each co-culture experiment. **(b**) Copy number profiles of cell lines utilized in co-culture experiments in **a)** and **c)**. **(c**) Absolute growth rates of WT 1q (blue) or +1q (red) aneuploid cells when co-cultured with either: diploid cells, WT 1q aneuploid cells, pre-1q gain isogenic ancestor cells, or +1q aneuploid cells. The left panel is without GSI, the right panel is + GSI (2 μM L-685,458). Log_2_FC growth rates relative to mono-culture are shown. *P* values calculated from two-sided *t*-tests. **(d**) Gene set enrichment plots for +1q-associated differential gene effect score rankings in breast cancer cells lines in the DepMap CRISPR (top) and RNAi (bottom) datasets using the curated Notch Activation gene set. *P* values and normalized enrichment scores (NES) calculated from GSEA. **(e**) Diagram illustrating potential implications of +1q Notch poising for tumor evolution. As +1q subclones emerge, they encounter mostly WT 1q cells and thus occupy fully Notch-ON states, providing growth advantage. As +1q cells take over, they run out of WT 1q cells to occupy Notch-OFF states and supply ligand and must occupy both Notch-ON and Notch-OFF states, diminishing the growth advantage. Source data

To test whether +1q HMECs can engage in dominant lateral inhibition when mixed with WT 1q cells as predicted by our model, we performed a series of co-culture experiments with blue fluorescent protein (BFP)- and crimson-tagged +1q or WT 1q HMECs. The +1q HMECs displayed increased Notch activation when co-cultured with WT 1q cells compared to mono-culture (Fig. 6f–h). The growth rate of +1q cells increases when engaged in dominant lateral inhibition with WT 1q cells, in a γ-secretase-dependent manner (Fig. 6i and Extended Data Fig. 10a–c). Analysis of DepMap CRISPR and RNA interference data revealed increased dependence on the Notch activation gene set in +1q cancer cell lines compared to WT 1q lines (Fig. 6j and Extended Data Fig. 10d). Taken together, we conclude that +1q is selectively beneficial in mammary epithelial cells via γ-secretase overexpression and Notch poising, which may confer especially strong selective advantage to +1q subclones in juxtracrine competition with WT 1q cells (Fig. 6k and Extended Data Fig. 10e). Of therapeutic relevance, γ-secretase inhibitors may alter these dynamics and could represent a targeted approach for +1q breast cancers.

## Discussion

In this study we performed unbiased chromosomal copy number genetic screens in normal human epithelial cells (mammary and renal). Recurrent selection of tissue-specific cancer-associated CNAs occurred in the absence of classical oncogene or tumor suppressor drivers in vitro. The isogenic aneuploid cell lines derived from our screens enabled exploration of the structural facilitators and genetic drivers of common cancer-associated CNAs. Interestingly, we observe fitness benefits from cancer-associated CNAs in the absence of *TP53* mutation. While p53 loss may enhance tissue-specific CNA fitness effects present inherently in certain cell types and accelerate/promote their acquisition^73,74^, it is not required for cancer-associated CNA selection in mammary or renal epithelial cells.

WGD accelerated karyotype evolution in HMECs, especially chromosomal loss and arm-level events. More investigation is required to determine whether the increase in arm-level event selection in tetraploids is due to increased basal rates of SV formation (due to increased replication stress^26,75^), increased tolerance for CNAs (due to smaller effect sizes) or increased selective pressure to attain beneficial CNAs, or combinations thereof. Whatever the causes, the consequences include increased access to evolutionary space and clonal heterogeneity.

Arm-level CNAs often arose as ‘paired’ two-copy events, structurally resolved through centromeric SVs to form isochromosomes or hybrid chromosomes. As centromeric translocations are one of the most frequent types of SV observed in human cancer and often associated with arm-level CNAs^76^, our in vitro system represents a good model for structural karyotype evolution in tumors.

In line with observations in other cell types^1,3,5^, most whole chromosome CNAs were detrimental to cellular fitness in HMECs and RPTECs, with some exceptions. However, convergent karyotypic evolution improved proliferation rates, coincident with reduced stress signatures. This suggests that specific CNAs can mitigate general aneuploidy stress even when they add to the total aneuploidy burden. Highly recurrent cancer-associated CNAs (+8q and +20) could even accelerate proliferation of diploid HMECs. Thus, both stress-reduction and pro-proliferative/survival effects probably contribute to the fitness benefits of cancer CNAs.

Our data support MYC as a driver of +8q, and propose Notch signaling as a driver of +1q via overexpression of 1q-resident γ-secretase genes. Increased Notch activation capacity tips lateral inhibition dynamics in favor of +1q cells occupying Notch-on states. This could potentially explain the dominance of +1q cells during in vitro evolution experiments, sometimes via parallel evolution of competing +1q subclones—a phenomenon noted in tumors^53^ and in adjacent normal mammary epithelia^55^. While in principle the Notch-poised state might not provide proliferative advantage after achieving a clonal +1q sweep (a concept illustrated in Extended Data Fig. 10e), it may continue driving growth at an invasive edge.

Altogether we show that cancer-associated CNAs can improve cellular fitness in untransformed epithelial cells independent of driver mutations via distinct structural and functional mechanisms, which may underlie tissue-specific CNA selection patterns during tumorigenesis.

## Methods

### Ethics declaration

The authors have complied with all ethics guidelines and have no competing interests to declare.

### Establishing clonal aneuploid cell lines

The hTERT–RPTEC cell line was purchased from ATCC (CRL-4031), and the hTERT–HMEC cell line was immortalized previously in the Elledge lab from primary HMECs purchased from ATCC (PCS-600-010). HEK293T cells were purchased from ATCC (CRL-3216). Low-passage hTERT–HMECs^78,79^ were grown in Lonza HMEC medium with bovine pituitary extract and growth supplements, and hTERT–RPTECs^80^ were grown in Gibco Dulbecco’s modified Eagle medium F12 with 2% fetal bovine serum and ATCC RPTEC growth supplements. DNA- and RNA-seq analysis on both cell lines utilized in this study confirmed cell type identity (please refer to Extended Data Fig. 1). A total of 1 × 10^6^ cells were treated with reversine (75 nM for HMECs and 150 nM for RPTECs) for 48 h, then split and allowed to recover without reversine for an additional two PDs. Single cells were plated in 384-well dishes in their respective medias (RPTEC media was supplemented with hypoxanthine and thymidine). Once at confluency, clones were transferred to 24-well plates and then to six-well plates and finally to 10-cm plates containing their respective media. Once clones reached confluency in the 10 cm dishes, they were trypsinized and approximately 20% of the cells were aliquoted each for DNA library preparation and propidium ioidide (PI) staining, and the remainder was frozen and banked in liquid nitrogen. Replicate screens were performed without PI staining or cryo-banking, as cells were lysed directly in 96-well plates after clonal seeding and outgrowth to collect DNA. For in vitro evolution experiments, cells were cultured in six-well dishes, with maximum density of ~1.5 × 10^6^ cells, split to ~1 × 10^5^ cells at each passage.

### PI staining for total DNA content

Approximately 5 × 10^5^ cells per clone were fixed in 70% ethanol, then stored for up to 1 month at −20 °C. Fixed cells were spun down, fixative was removed and then cells were washed once in phosphate-buffered saline (PBS) and finally resuspended in 500 μl Thermo Fisher FxCycle PI/RNAse staining solution. After incubation in the dark for 30 min, cells were passed through a mesh filter sieve and analyzed by fluorescence-activated cell sorting (FACS) using 532-nm excitation with a 585/42-nm bandpass filter. An average of 1 × 10^4^ events were analyzed per clone, with data collected via BD FACSDiva software v.8.0 and processed using FlowJo v8.8.6 to derive the average fluorescence of the G1 peak relative to that of diploid control cells processed simultaneously.

### Microscopy and image analysis

Cells were imaged in six-well plates using an inverted Zeiss bright field microscope at 20× magnification. For cell size and shape analysis, images were inverted and contrast was increased in Adobe Photoshop v18.1.6, then analyzed using CellProfiler v2.2.0 (ref. ^81^) using the following functions: (1) smooth, (2) IdentifyPrimaryObjects, (3) MeasureObjectSizeShape and (4) ExportToSpreadsheet.

### gDNA library preparation and sequencing

Genomic DNA (gDNA) was collected from a pellet of approximately 5 × 10^5^ cells per clone. Cells were lysed in 200 μl lysis buffer (10 mM Tris–HCl pH 8, 10 mM EDTA, 0.5% SDS, 0.75 mg ml^−1^ Proteinase K) and incubated overnight at 55 °C. Sodium chloride was added to a final concentration of 0.2 M and DNA was extracted with an equal volume of phenol/chloroform (UltraPure phenol:chloroform:isoamyl alcohol, 25:24:1 v/v), then samples were spun down and aqueous phases removed. To the aqueous phase, RNase was added to a final concentration of 25 μg ml^−1^ and samples were incubated overnight at 37 °C, then extracted again with phenol/chloroform. DNA was ethanol precipitated, dried and resuspended in DNase-free H_2_O. One microgram of gDNA was used as input for high-throughput sequencing library preparation. gDNA was sheared using NEB fragmentase enzyme mix at 37 °C for 35 min on a thermocycler, then the fragmented gDNA (approximated 200–300 bp fragments) was immediately purified with AmpureXP beads (1.5× volume). DNA ends were blunted and A′ tailed utilizing a mixture of 1× T4 ligase buffer containing ATP, 10 mM dNTPs, T4 DNA polymerase, T4 polynucleotide kinase and Taq DNA polymerase, as previously described^82^, incubating for 20 min at 25 °C, then 20 min at 72 °C on a thermocycler. To this reaction, T4 ligase was added, followed by 1.25 μl of NEBNext adaptor (diluted 2×); then the well-mixed samples were incubated at 20 °C for 15 min. A total of 1.5 μl of NEB User enzyme was added to each reaction, mixed well and incubated for 15 min at 37 °C. AmpureXP beads were added to each reaction (0.74× volume) to purify clean-up, adapter-ligated DNA fragments. Eluted DNA was polymerase chain reaction (PCR)-amplified for ten cycles using NEB index primers. A final round of DNA purification was done using the AmpureXP beads (0.9× volume) and gDNA libraries were eluted in 15 μl 0.1× TE buffer. Library concentrations were determined by nanodrop and multiplexed accordingly, then sequenced on a NextSeq500 (Illumina; Sequencing: Harvard Biopolymers Facility Genomics Core’s pipeline for NextSeq550 data acquisition; 2017–2021), high-output mode, single-end, 83 cycles plus 8 for the index, with 10% PhiX spike-in. Approximately 1–5 × 10^6^ reads per library were sequenced and used for copy number analysis. For deep-coverage WGS, 1 μg of gDNA was used to prepare PCR-free TruSeq DNA libraries. Library construction was done in accordance with the manufacturer’s protocol. The libraries were sequenced (paired-end, 150 cycles) on HiSeq-X (Illumina) machines with target coverages of 40× for the parental HMEC population, single-cell derived lineages (parental clones *ae*, *bq*, *CQ* and *BF*) and other derivative tetraploid clones (*CQ-ev-B*, *CQ-ev-D*, *CQ-ev-H*, *CQ-ev-L*, *CQ-ev-R*, *CQ-ev-T*, *FX*, *FF*, *FX-ev1-A*, *FX-ev1-B*, *FX-ev2-A* and *FX-ev3-A* clones), and 20× for diploid-range clones derived from *ae* (*ae-ev-a*, *ae-ev-b*, *ae-ev-c* and *ae-ev-f*) and *bq* (*bq-ev-a*, *bq-ev-b*, *bq-ev-c* and *bq-ev-d*). For single-cell sequencing immediately post-reversine treatment, single cells were sorted into 5 μl single cell lysis buffer and proteinase K-treated for 1 h at 55 °C, then whole genome amplified using the GenomePlex Single Cell Whole Genome Amplification Kit from Sigma (WGA4). The amplified gDNA was then converted into sequencing libraries using the adapter ligation and barcoding methods described above, then sequenced on a NextSeq500 (single-end, high-output 75 cycles).

### CNA calling from low-coverage DNA sequencing

Reads were aligned from fastq files to the human GRCh37 reference genome using the Burrows–Wheeler Alignment BWA^83^ v0.7.17 MEM function (default settings) and sorted using the SAMtools^84^ v1.3.1 sort function to generate sorted binary alignment map files. These files were used as input for a workflow in R based on the AneuFinder^85^ v1.22.0 findCNVs function. First, reads were binned into 500 kb bins, with any bins from problematic regions like centromeres and acrocentric short arms masked. An additional filter was applied to remove outlier bins on a per-chromosome basis. Then, the AneuFinder findCNVs function was applied to the binned data using the hidden Markov model (with baseline ploidy determined from PI staining for each clone used to seed the model). This function generates an aneuHMM object containing the binned data, breakpoint calls and copy number calls in the form of segment files. Segments were filtered using the filterSegments function such that the minimal segment width was 10 Mb, since the low-coverage sequencing data are too sparse to detect smaller segments. Since the AneuFinder model forces copy number calls into integer states, and our data occasionally consisted of subclonal populations, we added a subclone correction step to adjust copy number segments that differed appreciably from average bin read depth to accommodate average population intermediate copy number states.

### Read mapping and variant calling from deep-coverage DNA sequencing datasets

FASTQ files were aligned to human genome version GRCh37d5 (reference with decoy sequences; human_g1k_v37_decoy.fasta.gz) using the BWA v0.7.15 MEM function. PCR duplicates were marked using Picard tool v2.8.0 and indel realignment and base quality score recalibration were done by the Genome Analysis Toolkit, in accordance with the best practice pipeline (version 3.7). Pre-existing base substitutions and short indels in HMEC parental line were called by HaplotypeCaller function in the Genome Analysis Toolkit with default setting. Single nucleotide polymorphisms (SNPs) in general population was annotated using ANNOVAR software^86^ (version release of 2018-04-16), and the variants with minor allele frequency greater than 0.001 were considered as germline polymorphisms. Newly acquired base substitutions and indels were called by MuTect2 (ref. ^87^) for all clones separately, using parental line as paired reference. To precisely determine the presence or absence of the somatic mutations in our clones, we counted base compositions in all genomic positions where somatic mutation was called in at least one clone, using SAMtools software (version 1.3.1; mpileup function). Based on this result, phylogenic relationship between different clones was determined. SVs were detected using Delly^88^ v1.0 and SvABA^89^ v0.2.1 in their somatic calling pipelines with the parental HMEC population as the reference. For the Delly output, we started from the SVs with more than three supporting reads. After filtering out the SVs in the blacklist region listed in SV blacklist (available at ref. ^90^), all SVs were examined using Integrative Genomics Viewer (version 2.4.9)^91^ and false positive calls were filtered out. For the SvABA output, we used somatic output file (*.svaba.somatic.sv.vcf) for downstream analysis, after similar filtering process as Delly output. The filtered call sets were merged into a union set, and all the breakpoint locations were inspected in all sequenced clones to determine their presence. The phylogeny tree inferred from shared and private SVs was concordant with the one based on base substitutions.

### Mutational signature analysis

We classified base substitutions into 96 groups based on base exchange spectra (pyrimidine base as reference; C > A, C > G, C > T, T > A, T > C and T > G) and their adjacent nucleotide context (both 5′ and 3′ sides). Given the moderate number of newly acquired mutations and their spectrum obviously indicating large contribution of in vitro culture-associated mutations, we analyzed mutational signatures by expressing the observed spectrum in terms of linear combinations of the known mutational signature catalog^43^. Mutational spectra of all newly acquired mutations was decomposed in a linear combination of SBS1, SBS2, SBS5, SBS13, SBS17 and SBS18. Then, we assigned the exposure of each signature to the branches of our phylogeny tree with non-negative least squares algorithm using the NNLS R package v1.2-0. The decomposition was carried out for each clone. For the branches in the phylogeny, the exposures were distributed on the basis of the fraction of substitutions attributed to the branch, because we found no significant change in mutational spectra during the evolution experiment. The exposure of each signature was scaled by the ratio of the number of substitutions in that branch divided by the total number of substitutions in the clone. For a branch shared in the phylogeny of multiple clones, the exposure of each signature was calculated for all the clones that originate from the branch. The average of exposures for the signatures determined for all the related clones was taken as the final exposure of the signature in that branch of the phylogeny.

### Allele-specific CNA

To analyze allelic copy number of genomic segments, we utilized Sequenza^92^ with default settings. To determine allelic concordance in commonly gained chromosomal arms (chromosomes 1q and 20), we utilized heterozygous SNP site information stored in ‘.seqz’ intermediate files. All sites marked as ‘het’ were extracted from all clones with deep WGS. Then, we established a union SNP set by merging all the heterozygous SNP sites from different clones and calculated fraction of concordant major (A) alleles between all clonal combinations. This result was visualized in heatmaps using R package ComplexHeatmap v1.10.2.

### Correlation between genomic variants and epigenomic features

We studied correlation between SV breakpoints detected from deep WGS and various epigenomic features of mammary epithelial cells. We created a pseudo-vcf file including all SV breakpoint positions with randomly generated base substitutions and used this file as input for Mutalisk software^93^. We performed goodness of fit tests to assess if the distribution of the SV breakpoints is significantly different from the expected proportions of each epigenomic variable in the GRCh37. Chi-squared tests were used to determine the statistical significance. We used HMEC as reference epigenome for all analyses, except for replication timing, because this feature was unavailable for HMEC and instead we used replication timing information from MCF7 breast cancer cell line.

### Hi-C SV detection

Unsynchronized cells were trypsinized, resuspended and fixed in 2% formaldehyde, washed and 1 × 10^6^ cells were aliquoted, pelleted and stored at −80 °C for up to 1 month. Proximity-labeled gDNA was prepared from frozen fixed cell pellets essentially following the Arima Hi-C library preparation kit protocol. DNA was fragmented on a Covaris M220 using factory settings to achieve 400-bp fragments. Size selection was achieved with AmpureXP beads, followed by biotin enrichment according to Arima guidelines. End repair, A′-tailing and adapter ligation was done using KAPA Hyper Prep kit components, following Arima guidelines for use with bead-bound DNA, with Illumina TruSeq sequencing adapters used for indexing. After bead elution, libraries were amplified by PCR for 10 cycles and cleaned up with AmpureXP beads. Hi-C libraries were quantified using a Qubit fluorometer and dsDNA HS Assay Kit and multiplexed accordingly. Although we explored using longer reads up to 150 bp, we found that short 40-bp paired-end reads were sufficient to robustly map Hi-C interactions. All sequencing was performed on a NextSeq500 in high-output mode. An average of 2 × 10^7^ paired-end reads per sample library were sequenced, although we were able to map SVs for samples with as few as 5 × 10^6^ reads. Reads were aligned to the GRCh37 human genome using the BWA MEM^83^ version 0.7.15 with −SP settings to relax the proper pairing requirement to map distant and inter-chromosomal pairs generated by Hi-C. Generated binary alignment map files were then parsed into pairs files using pairtools v0.2.0 parse subcommand^94^ with the following settings: max-inter-align-gap = 80, max-molecule-size = 100,000,000, walks-policy = 5any and min-mapq = 1. These non-default settings were used to parse ‘walk’-like alignments, where ≥2 Hi-C fragments reside on one side of the paired-end read. The 1 Mb-binned cool files were generated from pairs files using the cooler^95^ v0.8.0 cload pairs function, then balanced to normalize copy number effects and other Hi-C-related biases using the cooler balance function, and from these files cooler dump was used to generate files with the frequencies of observed interactions. Observed interaction frequencies were normalized by the expected (normalized counts denoted as observed/expected (OE)), which were generated using cooltools v0.3.2 (ref. ^96^) compute-expected function. Intra-chromosomal (*cis*) expected was calculated as an average (per pixel) of interactions at a given genomic distance for each chromosome, while inter-chromosomal (*trans*) expected was calculated as an average of interactions for a given pair of chromosomes. A computational pipeline was developed to automatically detect trans-chromosomal fusions based on the HiNT^97^ algorithm but optimized for low-coverage sequencing. The OE values of 1 Mb × 1 Mb pixels across all inter-chromosomal regions were used to calculate four values: gini index 1 (gini inequality score based on the number of pixels with OE >3 across 1 Mb columns of the inter-chromosomal heatmap), gini index 2 (gini inequality score based on the number of pixels with OE values >3 across 1 Mb rows of the inter-chromosomal heatmap), entire inter-chromosomal gini index (based on all OE scores for each pair of chromosomes) and a maximum OE score that takes the average OE value for the five pixels with greatest OE values. A combination score for each inter-chromosomal arm versus arm region was generated on the basis of these scores, then normalized to the respected combination score from a diploid control. Inter-chromosomal arm versus arm regions with high scores after normalization indicate translocations. Genome-wide interaction plots for each sample were also manually inspected to detect translocations, and, in the vast majority of cases, manual inspection calls agreed well with computationally predicted translocation calls. If calls disagreed, we deemed a translocation uncertain and removed it from downstream meta-analysis. Isochromosomes could not be directly detected by Hi-C, so Giemsa staining (performed by the Brigham and Women’s Hospital Cytogenomics Core) was employed to validate suspected isochromosomes. Two out of two putative isochromosome-containing lines in the Giemsa validation set could be validated.

### TCGA analysis

Level 3 genome-wide copy number and transcriptomic data from TCGA Research Network^98^ was downloaded using the Broad GDAC firehose (http://gdac.broadinstitute.org/). The specific data types used were SNP array-based segmented copy number (minus germline) files for CNA calling and RNA-seq by expectation maximization normalized files for gene expression analysis. To determine samples with whole chromosome and arm-level chromosome CNAs we first corrected the copy number log_2_ segment mean scores based on previously calculated tumor purity estimates^99^. For a log_2_-transformed copy number ratio *x*, and tumor purity fraction *p*, we derived a purity-corrected log_2_-transformed copy number ratio *c*:

$$c=\log_2\left(\frac{{2}^{x}-(1-p)}{p}\right)$$

Gains were called for purity-corrected segment mean greater than 0.32, and losses were called for purity-corrected segment mean less than −0.415. These thresholds correspond to gain or loss of at least one copy in a pure tetraploid population, or gain or loss of one copy in at least half of a diploid tumor population. If all gain or loss segments cumulatively spanned at least 75% of a whole chromosome, or 50% of a chromosome arm, depending on the analysis type, we called that chromosome region gained or lost. The tumor types used for comparisons to our in vitro data are the 10 most common and/or most deadly tumor types for men and women in the United States, according to National Cancer Institute’s Surveillance, Epidemiology and End Results program and the Centers for Disease Control and Prevention’s National Program of Cancer Registries^100^, which were represented by at least 100 samples in the TCGA database. We excluded leukemias and thyroid cancer due to a general lack of aneuploidy. We included related tumor site subtypes (when available in the TCGA) as separate cohorts (that is colon and rectal cancer, kidney clear cell and kidney papillary, and lung adenocarcinoma and squamous cell carcinoma). PAM50 messenger RNA signatures were used to define breast cancer molecular subtypes for Extended Data Fig. 9c, but for most analyses all breast cancer subtype data is pooled^101^. Differential gene expression tests among various CNA-subsetted cohorts were performed using glmFIT and glmRT functions from the edgeR package^102^. Signed negative log_10_ *P* values were used to rank gene lists for GSEA analysis^103^, which was performed in weighted mode using the Hallmarks gene sets with 1,000 permutations.

### PCAWG breast cancer analysis

We downloaded the processed datasets of Pan-Cancer Analysis of Whole Genomes (PCAWG) consortium from the International Cancer Genome Consortium Data Portal (http://dcc.icgc.org). We identified a total of 208 breast cancer cases with available base substitution, copy number variation and SV information, including 129 ductal adenocarcinomas, 13 lobular adenocarcinomas and 3 ductal carcinomas in situ. We utilized WGD status determined by the consortium and analyzed the burden of genomic variants between the tumors with and without WGD. The number of each class of variants, including base substitutions, indels and SVs, corrected by ploidy estimates, were compared using Student’s *t-*test. Copy number profile of individual tumors were piled up together for both groups of tumors with and without WGD, using custom R code for graphical presentation.

### CCLE and DepMap analysis

RNA-seq and copy number data for cancer cell lines from the CCLE^77^ were downloaded through the DepMap portal^104^. Since the purity complications that arise in human tumor sample data were not present in the cell line data, we simply correlated 1q copy number status with gene expression rather than choose a cutoff for +1q gain/loss and partition into groups. We correlated each gene’s expression with the average copy number of *APH1A*, *NCSTN* and *PSEN2*, the three γ-secretase genes on 1q. The direction and significance of the correlation for each gene with 1q copy number were used to rank genes based on how up- or down-regulated they were in conjunction with 1q status. CERES-corrected combined CRISPR data and the combined RNAi screen data^105–107^, acquired through the DepMap portal, were used to correlate gene effect scores with the average copy number of *APH1A*, *NCSTN* and *PSEN2*.

### Growth assays

A total of 2 × 10^4^ cells were plated in 24-well plates in at least triplicate per cell line. The following day after plating, cells were counted with an automated cell counter, and this count served as the baseline ‘day 0’ count for each replicate to account for differences in plating efficiency. Cells were counted each day for 5 days or until nearly confluent, with media being refreshed on day 3. Time course data were fit to a simple exponential growth model to derive growth rates, since we did not observe substantial deviations from constant growth during the course of the experiments.

### RNA-seq library preparation and analysis

A total of 2 × 10^5^ cells from each cell line were plated in six-well plates and grown for 48 h. Cells were provided fresh media 3 h before collecting. Media was aspirated and cells were immediately lysed in dishes and total RNA was purified using Qiagen RNeasy kits. A quantity of 1 μg of total RNA was used for mRNA purification with the NEBNext Poly(A) mRNA Magnetic Isolation Module. NEBNext Ultra II Directional RNA Library Prep Kits for Illumina were used for RNA-seq library preparation. NEBNext Multiplex Oligos for Illumina were used for indexing during PCR amplification of the final libraries. Libraries were quantified by nanodrop and multiplexed accordingly. Sequencing was performed on a NextSeq500, high-output mode, single-end for 83 cycles plus 8 for the index, with 10% PhiX spike-in. Reads were aligned to the GRCh37 human genome annotated with gencode gene sets (version 32)^108^, using the BWA algorithm with default settings^83^. An average of 6.5 × 10^6^ reads were aligned per sample (range of 4.5−8.0 × 10^6^). Read counts per gene were calculated using the featureCounts function from the Subread package v1.6.2 (ref. ^109^). Differential gene expression was performed using the glmFIT and glmRT functions from the edgeR package v3.36.0 (ref. ^102^), with a minimum reads per kilobase per million mapped reads of 2. Signed negative log_10_ *P* values were used to rank gene lists for GSEA analysis using fgsea v1.20.0 (ref. ^103^), which was performed in weighted mode using the Hallmarks gene sets with 10,000 permutations, unless otherwise noted.

### Notch activation assay

A total of 2 × 10^5^ cells from each HMEC line indicated in Fig. 6e were plated in six-well plates and grown for 48 h. One arm of the experiment was pre-treated with 100 nM GSI (Abcam cat. no. ab145891) for 30 min before EGTA treatment. The pre-treated GSI arm and another non-pre-treated arm were then washed with PBS and incubated for 10 min in PBS and 4 mM EGTA for 10 min at 37 °C. The untreated arm was kept in regular medium. After EGTA incubation, all three arms of the experiment were lysed immediately in the wells with 300 µl 2× RIPA buffer (Boston Bioproducts cat. no. BP-115X) plus protease inhibitor cocktail (Fisher cat. no. 78440). Lysates were vortexed and spun down, and protein concentrations were determined by bicinchoninic acid protein assay (Pierce cat. no. 23227), then equal amounts of protein were mixed with lithium dodecyl sulfate sample buffer (Invitrogen cat. no. NP0007) and loaded onto 4–12% Bis-Tris gels, 1.5 mM, with 15 wells (Invitrogen cat. no. NP0336BOX). Gels were run in MOPS SDS buffer (Life Technologies cat. no. NP0001) and transferred to nitrocellulose (BioRad cat. no. 170-4158), blocked overnight in 3% BSA at 4 °C, then incubated overnight at 4 °C with N1ICD antibody (Cleaved Notch1 (Val1744) (D3B8) rabbit mAb, Cell Signaling cat. no. 4147S) at 1/500 dilution in TBST buffer with 1% BSA, or with NCSTN antibody (Nicastrin (D4F6N) rabbit mAb, Cell Signaling cat. no. 30239S) at 1/1,000 dilution, or with GAPDH antibody (GAPDH (D16H11) XP Rabbit mAb, Cell Signaling cat. no. 5174S) at 1/10,000 dilution. Secondary antibody for all assays was goat anti-rabbit IgG (Abcam cat. no. ab205718), incubated at 1/10,000 dilution for 1 h at room temperature. Western blots were quantified using ImageJ v1.53a, and N1CD or NCSTN values were normalized to GAPDH values.

### CRISPR knockdown of *NCSTN*

*NCSTN*-targeting sgRNAs were cloned into the lentiCRISPR v2 backbone and packaged into lentivirus via transfection into HEK293T cells along with third-generation lentiviral packaging vectors. Lentivirus was collected and used to infect either diploid parental or +1q HMECs. Infected cells were selected with 2 μg ml^−1^ puromycin for 2 days. Population-level NCSTN protein reduction was quantified via western blot using a NCSTN antibody (Nicastrin (D4F6N) rabbit mAb, Cell Signaling cat. no. 30239S at 1/1,000 dilution) and normalized to GAPDH staining. Guide RNA sequences are as follows: *AAVS1* loci: GGGGCCACTAGGGACAGGAT, *NCSTN* sg1: GTCACTGCAGAGAAATACAG, and *NCSTN* sg2: GTAGGACGCAGAAAGACAGA.

### Notch modeling

We implemented the Notch signaling model described previously^72^ with the following alteration to the equation describing Notch activation:

Original equation:

$$\frac{{{\mathrm{d}}N}\,}{{{\mathrm{d}}t}}={\mu }_{N0}\left(-(1+{F}_\mathrm{p})N+\frac{{\left(\frac{{\sum }_{\mathrm{neighbors}}{D}_\mathrm{p}}{{K}_{D}}\right)}^{2}}{1+{\left(\frac{{\sum }_{\mathrm{neighbors}}{D}_\mathrm{p}}{{K}_{D}}\right)}^{2}}\right)$$

Modified equation:

$$\frac{{{\mathrm{d}}N}\,}{{{\mathrm{d}}t}}={\mu }_{N0}\left(-(1+{F}_\mathrm{p})N+\gamma \left(\frac{{\left(\frac{{\sum }_{\mathrm{neighbors}}{D}_\mathrm{p}}{{K}_{D}}\right)}^{2}}{1+{\left(\frac{{\sum }_{\mathrm{neighbors}}{D}_\mathrm{p}}{{K}_{D}}\right)}^{2}}\right)\right)$$

The modification introduces a constant scaling factor *γ* that represents the degree of Notch poising. Additionally, we collapsed the two Hes factors utilized in Sancho et al. into one term. All simulations were performed in a 40 × 40 matrix of hexagonal cells. Simulations were initiated with random values for each cell in the matrix between 0 and 1 for the terms *N*, *D*_m_, *F*_m_, *D*_p_ and *F*_p_, or between 0 and 0.1 for *H*_m_ and *H*_p_ terms. The outer rim of the field of cells was kept fixed at the initial random values, while all other cells were allowed to change over time. Constant values including *μ* and *K* terms were kept the same as Sancho et al. values, with the following exceptions: *μ*_*D*m_ = 0.01, *v* = 30.

### Co-culture transcriptional assays

BFP- and crimson-expressing HMEC lines were generated by lentiviral infection using pHAGE–EF1-dest–tagBFP or pHAGE–EF1-dest–E2C vectors at an multiplicity of infection of approximately 0.5, followed by FACS-based sorting of the BFP+ or crimson+ populations. To assay transcriptional effects of mixing +1q and WT 1q populations, we co-cultured red +1q and blue WT 1q (and vice versa for the color swap) cell lines in the following manner: 1 × 10^5^ +1q cells and 1 × 10^5^ WT 1q cells of opposite color were mixed and plated per well in six-well dishes, each well containing a different match-up of individual lines (three WT 1q lines versus four +1q lines, 12 different combinations). Reciprocal color-swap experiments were also set up. Controls consisted of red and blue versions of the same line mixed together. After 72 h, cells were trypsinized in the presence of 4 μM GSI DAPT (Sigma cat. no. D5942-5MG) to prevent acute activation of Notch via trypsinization, pooled according to the experimental arm, and sorted by color. Sorted cells were pelleted and RNA was collected and sequenced as described above. Data were analyzed by comparing co-cultured cells to their respective control mono-cultured cells, using edgeR and GSEA as described above.

### Competition assays

Using the BFP- and crimson-tagged cell lines described above, we mixed and plated 2 × 10^4^ blue and 2 × 10^4^ red cells in each well of a 24-well plate. Each well contained a different combination of cell lines (all-by-all matrix of six WT 1q lines, two pure diploid lines, and five +1q lines −78 different combinations, see Extended Data Fig. 10a). The reciprocal color-swap experiments were also set up. In one arm of the experiment, 2 μM GSI (L-685,458, Abcam cat. no ab141414) was added to the wells upon cell plating. After 72 h in culture, the fractions of red/blue cells in each well were measured in the control and +GSI conditions via FACS. FACS data were analyzed using the flowCore v2.6.0 (ref. ^110^) and ggcyto v1.22.0 (ref. ^111^) R packages. For every cell line combination, we derived the change in the crimson fraction in the +GSI versus control conditions. Plotted in Extended Data Fig. 10a is the average of three biological replicates. This experiment is summarized in Fig. 6g, where we collapsed +1q or WT 1q cell lines each into one group. We repeated this general experimental setup with a smaller subset of cell lines for Extended Data Fig. 10c but plated more cells (1 × 10^5^ per cell line, 2 × 10^5^ total) in six-well dishes and included counting beads (CountBright Absolute Counting Beads, Thermo Fisher cat. no. C36950) during FACS assays to determine total cell counts. This enabled us to estimate growth rates of each cell line in in the co-culture experiment.

### Statistics and reproducibility

All comparative data analysis was performed using standard statistical methodologies and internal experimental controls. No statistical method was used to predetermine sample size; sample sizes for each experiment were maximized on the basis of experimental feasibility and sample availability, with most experiments including multiple independently derived cell lines as biological replicates. No data were excluded from the analyses. The experiments were not randomized. The investigators were not blinded to allocation during experiments and outcome assessment. All boxplots include the following: upper and lower limits of box plot—first and third quartiles, middle bar of box plot—median, and upper and lower whiskers—extend to the largest/smallest value no further than 1.5 times the interquartile range from those limits.

### Reporting summary

Further information on research design is available in the Nature Portfolio Reporting Summary linked to this article.

## Online content

Any methods, additional references, Nature Portfolio reporting summaries, source data, extended data, supplementary information, acknowledgements, peer review information; details of author contributions and competing interests; and statements of data and code availability are available at 10.1038/s41588-024-01665-2.

### Supplementary information

Supplementary Information Supplementary legends.

Reporting Summary

Supplementary Tables Supplementary Table 1. Acquired mutations during in vitro evolution experiments across all sequenced clones. Supplementary Table 2. Mutations present in parental hTERT–HMEC population, from which all clones were derived. Supplementary Table 3. All SVs acquired during in vitro evolution experiments across all sequenced clones. Supplementary Table 4. Curated Notch activation and repression gene sets.

Supplementary Video Simulation of Notch-on/Notch-off pattern formation in 40 × 40 cell lattice. Left: WT 1q control mono-culture experiment (50% WT 1q plus 50% WT 1q homogeneous population). Middle: WT 1q versus +1q co-culture experiment (50% WT 1q plus 50% +1q mixed population). Right: the +1q mono-culture experiment (50% +1q plus 50% +1q homogeneous population).

### Source data

Source Data Figs. 1–6 and Extended Data Figs. 1–10 All statistical source data, unprocessed western blots, raw cell image data and G-banding karyotype images. Supporting all main figures (Figs. 1–6) and all Extended Data figures (Extended Data Figs. 1–10).

## Data Availability

Sequencing data are available in the Sequence Read Archive (SRA; NCBI/NLM) under accession number PRJNA634423. Source data are provided with this paper.

## Code availability

Code written for this project is available on GitHub (https://github.com/emmavwatson). Code repositories: CNAplot v1.0 (ref. ^112^), CNorm v1.0 (ref. ^113^), SparseHiC v1.0 (ref. ^114^) and NotchModel v1.0 (ref. ^115^). Code organized by figure with accompanying RData files can be found in the NatGen2024 v1.0 repository^116^.
