# Supplementary material for: Chromosome evolution screens recapitulate tissue-specific tumor aneuploidy patterns
Source: Nat Genet. 2024 Feb 22;56(5):900–12. doi: 10.1038/s41588-024-01665-2 (PMC11096114; doi:10.1038/s41588-024-01665-2)
Supplement: Supplementary file 1 — Supplementary legends. [file 41588_2024_1665_MOESM1_ESM.pdf]

nature genetics

Article

https://doi.org/10.1038/s41588-024-01665-2

Chromosome evolution screens recapitulate tissue-specific tumor aneuploidy patterns

In the format provided by the authors and unedited

Supplementary Table 1 Acquired mutations during *in vitro* evolution experiments across all sequenced clones.

Supplementary Table 2 Mutations present in parental hTERT-HMEC population, from which all clones were derived.

Supplementary Table 3 All SVs acquired during *in vitro* evolution experiments across all sequenced clones.

Supplementary Table 4 Curated Notch Activation and Repression gene sets.

Supplementary Movie 1 Simulation of Notch-ON/Notch-OFF pattern formation in 40x40 cell lattice. Left panel: WT 1q control mono-culture experiment (50% WT 1q + 50% WT 1q homogenous population). Middle panel: WT 1q vs +1q co-culture experiment (50% WT 1q + 50% +1q mixed population). Right panel: +1q mono-culture experiment (50% +1q + 50% +1q homogenous population)
