# Supplementary material for: Chromosome evolution screens recapitulate tissue-specific tumor aneuploidy patterns
Source: Nat Genet. 2024 Feb 22;56(5):900–12. doi: 10.1038/s41588-024-01665-2 (PMC11096114; doi:10.1038/s41588-024-01665-2)
Supplement: Supplementary file 2 — Reporting Summary [file 41588_2024_1665_MOESM2_ESM.pdf]

nature portfolio

Corresponding author(s): Stephen J. Elledge and Peter J. Park

Last updated by author(s): 03/16/2022

Reporting Summary

Nature Portfolio wishes to improve the reproducibility of the work that we publish. This form provides structure for consistency and transparency in reporting. For further information on Nature Portfolio policies, see our Editorial Policies and the Editorial Policy Checklist.

Statistics

For all statistical analyses, confirm that the following items are present in the figure legend, table legend, main text, or Methods section.

n/a	Confirmed	
☐	☒	The exact sample size (*n*) for each experimental group/condition, given as a discrete number and unit of measurement
☐	☒	A statement on whether measurements were taken from distinct samples or whether the same sample was measured repeatedly
☐	☒	The statistical test(s) used AND whether they are one- or two-sided<br>*Only common tests should be described solely by name; describe more complex techniques in the Methods section.*
☐	☒	A description of all covariates tested
☐	☒	A description of any assumptions or corrections, such as tests of normality and adjustment for multiple comparisons
☐	☒	A full description of the statistical parameters including central tendency (e.g. means) or other basic estimates (e.g. regression coefficient) AND variation (e.g. standard deviation) or associated estimates of uncertainty (e.g. confidence intervals)
☐	☒	For null hypothesis testing, the test statistic (e.g. *F*, *t*, *r*) with confidence intervals, effect sizes, degrees of freedom and *P* value noted<br>*Give P values as exact values whenever suitable.*
☐	☒	For Bayesian analysis, information on the choice of priors and Markov chain Monte Carlo settings
☐	☒	For hierarchical and complex designs, identification of the appropriate level for tests and full reporting of outcomes
☐	☒	Estimates of effect sizes (e.g. Cohen's *d*, Pearson's *r*), indicating how they were calculated

Our web collection on statistics for biologists contains articles on many of the points above.

Software and code

Policy information about availability of computer code

Data collection	Flow cytometry: BD FACSDiva software v.8.0<br>Sequencing: Harvard Biopolymers Facility Genomics Core's pipeline for NextSeq550 data acquisition; 2017-2021
Data analysis	Flow Cytometry: FlowJo v8.8.6, flowCore v2.6.0, ggcyto v1.22.0<br>Image Analysis: Adobe Photoshop v18.1.6, CellProfiler v2.2.0, ImageJ v1.53a<br>Low Coverage DNA-seq: bwa v0.7.17, SAMtools v1.3.1, AneuFinder v1.22.0<br>High-Coverage DNA-seq: bwa v0.7.15, GATK v3.7, ANNOVAR version release of 2018-04-16, MuTect2 (within GATK), SAMtools v1.3.1, Delly, SvABA v0.2.1, Integrative Genomics Viewer v2.4.9, NNLS v1.2-0, Sequenza v2.1.2, ComplexHeatmap v1.10.2, Mutalisk (webtool, no version information available)<br>Hi-C analysis: bwa v0.7.17, pairtools v0.2.0, cooler v0.8.0, cooltools v0.3.2<br>Gene Expression Analysis: bwa v0.7.17, subread v1.6.2, edgeR v3.36.0, GSEA (fgsea v.1.20.0), CNorm v1.0<br>Additional code is available on E.V.W.'s github page (https://github.com/emmavwatson)

For manuscripts utilizing custom algorithms or software that are central to the research but not yet described in published literature, software must be made available to editors and reviewers. We strongly encourage code deposition in a community repository (e.g. GitHub). See the Nature Portfolio guidelines for submitting code & software for further information.

Data

Policy information about availability of data

All manuscripts must include a data availability statement. This statement should provide the following information, where applicable:

- Accession codes, unique identifiers, or web links for publicly available datasets
- A description of any restrictions on data availability
- For clinical datasets or third party data, please ensure that the statement adheres to our policy

The sequencing datasets generated for this study are deposited in SRA, under accession number of PRJNA634423
Human reference genome: GRCh37d5 (reference with decoy sequences); human_g1k_v37_decoy.fasta.gz
Tumor copy number and RNA-seq data: TCGA (https://gdac.broadinstitute.org/)
Tumor WGD information: PCAWG (http://dcc.icgc.org)

Field-specific reporting

Please select the one below that is the best fit for your research. If you are not sure, read the appropriate sections before making your selection.

☒ Life sciences ☐ Behavioural & social sciences ☐ Ecological, evolutionary & environmental sciences

For a reference copy of the document with all sections, see nature.com/documents/nr-reporting-summary-flat.pdf

Life sciences study design

All studies must disclose on these points even when the disclosure is negative.

Sample size	For TCGA analysis, all available samples were used that had data types of interest (i.e. Copy number, RNA-seq). For our in vitro copy number proliferation screens, we derived and sequenced ~100 aneuploid HMEC lines and 76 aneuploid RPTEC lines, which were the maximum numbers we could collect given our capacity in tissue culture. Replicate screens in both tissues revealed highly consistent results, indicating that we had collected sufficient clones to quantify CNA frequency in our cohorts. For our in vitro evolution experiments, we evolved >70 HMEC cultures over the course of approx. two months on average; again, this was the maximum number of experiments we could manage. For all RNAseq experiments and investigations of 1q+, no sample sizes were pre-determined based on sample size/power calculations. We collected on average three biological replicates per cell line for all RNA-seq experiments, which was sufficient to capture the direct effects of the copy number alterations on the transcriptomes of aneuploid cells in a statistically significant manor, indicating that we had collected sufficient numbers of samples to accurately reflect the transcriptomes of each aneuploid. For our investigations of +1q, we utilized on average 5 independently evolved cell lines with +1q and 5 independently evolved control lines (WT 1q), with a minimum of three biological replicates for each cell line/experimental condition. This reflects the maximum experimental capacity for these comparisons.
Data exclusions	no data were excluded from any analysis
Replication	We performed three biological replicates for the majority of experiments. Generally we had very high reproducibility between biological replicates for the same cell line/treatment.
Randomization	Most of our analyses involve comparisons of groups with different genomic feature status (i.e. +1q vs WT 1q), in these cases samples are assigned to groups based on the genomic feature of interest. Since copy number alterations sometimes co-vary in tumor cohorts, we previously developed an algorithm called CNorm (https://github.com/emmavwatson/CNorm) which attempts to control for copy number covariates by generating custom cohorts of patients with more even distributions of CNAs relative to a CNA of interest. This was used for the analysis in Fig. 2e. For comparative analyses within our isogenic cell lines, we could not utilize CNorm due to insufficient in vitro sample numbers, so co-variate CNAs could not be analyzed and correction was not implemented.
Blinding	Blinding was not relevant to study, as all experiments were set up and collected by a small group of individuals, who then also analyzed the data, thus it was not feasible.

Reporting for specific materials, systems and methods

We require information from authors about some types of materials, experimental systems and methods used in many studies. Here, indicate whether each material, system or method listed is relevant to your study. If you are not sure if a list item applies to your research, read the appropriate section before selecting a response.

Materials & experimental systems

n/a	Involved in the study
☐	☒ Antibodies
☐	☒ Eukaryotic cell lines
☒	☐ Palaeontology and archaeology
☒	☐ Animals and other organisms
☒	☐ Human research participants
☒	☐ Clinical data
☒	☐ Dual use research of concern

Methods

n/a	Involved in the study
☒	☐ ChIP-seq
☐	☒ Flow cytometry
☒	☐ MRI-based neuroimaging

Antibodies

Antibodies used

N1ICD antibody: Cleaved Notch1 (Val1744) (D3B8) Rabbit mAb Cell Signaling cat. # 4147S. 1/500 dilution.
GAPDH antibody: GAPDH D16H11 XP Rabbit mAb, Cell Signaling cat. # 5174S. 1/10,000.
NCSTN antibody: Nicastrin (D4F6N) Rabbit mAb, Cell Signaling cat. # 30239S. 1/1,000 dilution.

Validation

All antibodies were purchased commercially and authenticated by the manufacturer as described on their websites; antibodies have also been independently validated in multiple publications. We observed canonical behavior of Notch cleavage using the N1ICD antibody, and it consistently showed a single band at the correct MW. The GAPDH antibody is widely used and has been validated elsewhere, and was observed as a single band at the correct MW. We validated the NCSTN antibody with CRISPR-mediated knockdown.

N1ICD antibody: According to the manufacturer's website, "This antibody has been validated using SimpleChIP® Enzymatic Chromatin IP Kits... Validated for WB, WB, IHC, IF." There is also a validation experiment provided showing N1ICD binding via chromatin IP to canonical target HES4 in a g-secretase dependent manner. It has been cited by 479 journal articles, with links available on manufacturer's website (https://www.cellsignal.com/products/primary-antibodies/cleaved-notch1-val1744-d3b8-rabbit-mab/4147)

GAPDH antibody: According to the manufacturer's website, "Validated for WB, WB, IHC, IF." Western blot images showing GAPDH staining at the correct MW are shown. It has been cited by 6310 journal articles, with links available on manufacturer's website (https://www.cellsignal.com/products/primary-antibodies/gapdh-d16h11-xp-rabbit-mab/5174)

NCSTN antibody: According to the manufacturer's website, "Validated for WB, IP, IF, IF. Highly specific and rigorously validated in-house." Western blot images showing NCSTN staining at the correct MW are shown, as well as IF images showing correct expression/localization. We internally validated this antibody with two different CRISPR guides targeting NCSTN.

Eukaryotic cell lines

Policy information about cell lines

Cell line source(s)

The hTERT-RPTEC cell line was purchased from ATCC, and the hTERT-HMEC cell line was immortalized previously in the Elledge lab from primary HMEC cells purchased from ATCC. HEK293T cells were purchased from ATCC.

Authentication

Cell lines were authenticated by ATCC, but also we have shown by RNAseq analysis that they exhibit appropriate Kidney- or Breast-specific gene expression. We have also verified with various methods that both RPTEC and HMEC lines are diploid (46 chromosomes), and the RPTEC line is male, while the HMEC line is female.

Mycoplasma contamination

Both RPTEC and HMEC cell lines were tested in the lab for mycoplasma and both were negative.

Commonly misidentified lines
(See ICLAC register)

No commonly misidentified cell lines were used in the study.

Flow Cytometry

Plots

Confirm that:

☒ The axis labels state the marker and fluorochrome used (e.g. CD4-FITC).

☒ The axis scales are clearly visible. Include numbers along axes only for bottom left plot of group (a 'group' is an analysis of identical markers).

☒ All plots are contour plots with outliers or pseudocolor plots.

☒ A numerical value for number of cells or percentage (with statistics) is provided.

Methodology

Sample preparation

For PI staining of total DNA content: 5×10^5 cells per clone were fixed in 70% ethanol, then stored for up to 1 month at −20° C. Fixed cells were spun down, fixative was removed, and then cells were washed once in PBS and finally resuspended in

500uL ThermoFisher FxCycle PI/RNAse staining solution. After incubation in the dark for 30 min, cells were passed through a mesh filter sieve and analyzed by FACS using 532-nm excitation with a 585/42-nm bandpass filter. An average of 1×10^4 events were analyzed per clone.

For BFP- vs Crimson- co-culture RNA-seq experiments: we co-cultured red +1q and blue WT 1q (and vice versa for the color-swap) cell lines in the following manner: 1x10^5 +1q cells and 1x10^5 WT 1q cells of opposite color were mixed and plated per well in 6-well dishes. Controls consisted of red and blue versions of the same line mixed together. After 72 hours, cells were trypsinized in the presence of 4 uM g-secretase inhibitor DAPT (Sigma cat. # D5942-5MG) to prevent acute activation of Notch via trypsinization, pooled according to the experimental arm, and sorted by color.

For BFP- vs Crimson- co-culture growth assays: we mixed and plated 2x104 blue and 2x104 red cells in each well of a 24-well plate. After 72 h in culture, the fractions of red/blue cells in each well were measured in the control and +GSI conditions via FACs. We repeated this general experimental setup with a smaller subset of cell lines for Fig. S9c but plated more cells (1x105 per cell line, 2x105 total) in 6 well dishes and included counting beads during FACs assays to determine total cell counts. This enabled us to estimate growth rates of each cell line in mono-culture.

Instrument

For FACs measurements, the instrument used was a BD LSRII. For cell sorting, the instrument used was a Sony MA900

Software

We utilized FlowJo, flowCore , and ggcyto to analyzed FACs data.

Cell population abundance

For our co-culture growth assays, FACs counting beads (CountBright™ Absolute Counting Beads) were used to estimate total cells in each well. For cell sorting experiments, we confirmed purity based on downstream RNAseq data of populations.

Gating strategy

Gating strategies were utilized according to standard practice for flow cytometry. BFP and Crimson were chosen due to their low spectral overlap and we found that compensation was not necessary for our co-culture experiments.

☒ Tick this box to confirm that a figure exemplifying the gating strategy is provided in the Supplementary Information.
